# Knowledge, Attitudes and Practices Related to Medication, Antibiotics, and Vaccination among Public Service Population: National Survey Conducted in France

**DOI:** 10.3390/ijerph192114044

**Published:** 2022-10-28

**Authors:** Virginie-Eve Lvovschi, Florence Carrouel, Benjamin du Sartz de Vigneulles, Michel Lamure, Geneviève Motyka, Laurie Fraticelli, Claude Dussart

**Affiliations:** 1 Emergency Department, Rouen University Hospital, 76031 Rouen, France; 2 Laboratory “Research on Healthcare Performance” (RESHAPE), INSERM U1290, Université Claude Bernard Lyon 1, 69008 Lyon, France; 3 Hospices Civils of Lyon, 69003 Lyon, France; 4 Laboratory “Health, Systemic, Process” (P2S), UR4129, University Claude Bernard Lyon 1, University of Lyon, 69008 Lyon, France; 5 Caisse Nationale d’Assurance Maladie, 75020 Paris, France

**Keywords:** antibiotics, anxiolytic, health knowledge, attitudes, practice, hypnotic, polypharmacy, prevention, retired, vaccination hesitancy, worker

## Abstract

Medication, antibiotics, and immunization are three major and cost-effective medical interventions but their use is balanced. Knowledge, attitudes and practices (KAP) are a cornerstone. This retrospective study aims at analyzing KAP related to these concerns among the public service population in order to establish the basis for the implementation of selective preventive actions. From a cross-sectional anonymous online questionnaire-based survey among the insurees of a French mutual organization (*Union Prévention Santé pour la Fonction publique*, UROPS), 33 questions related to medication, antibiotics and vaccination were extracted to evaluate KAP. New variables were constituted: levels of knowledge, antibiotic misuse, proactive behavior and vaccinophobia. Multiple correspondence analysis was performed to identify respondents’ homogenous groups. In addition, bivariate statistical comparisons were provided and logistic regressions were carried out to identify determinants of these new variables. Public service population (workers and retired) were highly exposed to polymedication (8.7% vs. 24.4%, *p* < 0.0001), hypnotics overtake (24.3% vs. 18.4%, *p* < 0.0001), and misuse antibiotics (33.2% vs. 22.6%, *p* < 0.0001) despite good levels of knowledge. Proportions of vaccinophobia was low (0.8% vs. 1.7%, *p* < 0.0001). However, workers have different KAP than retired, without shared determinants in the 3 health domains studied. Respondents were proactive (85.8% vs. 81.6%, *p* < 0.0001), used multiple sources of trustworthy information and seems to be ready for the delegation of health tasks. Thus, preventive actions related to antibiotics and polymedication should be a priority in vaccination education for mutual organizations such as UROPS. Studying their insurees longitudinally could be interesting to highlight the impact of selective prevention on behaviors, through trusted health professionals (general practitioners, pharmacists…).

## 1. Introduction

Medication, antibiotics and vaccination are three of the most cost-effective life-saving medical interventions and have contributed to an extended lifespan from around 40 years old to more than 80 [1,2,3,4]. Today, the context of their use is more complex, and their effect is less clear-cut. Polymedication [5,6] and the high use of psychotropic drugs (anxiolytics and hypnotics) are significatively associated with a decrease in the health-related quality of life, regarding physical or mental status [6,7,8]. Antibiotic resistance [9,10] due to the misuse of antibiotics is increasing and has led to 700,000 deaths globally per year [2,11], and antibiotic sparing is included in the recommendations of the surviving sepsis campaign [12]. Hence, in primary care research, topics such as the rational use of drug prescriptions [13,14], particularly antibiotics and hypnotics [15,16], have become priority research topics worldwide [17,18]. Vaccine hesitancy, defined as the refusal or delayed acceptance to be vaccinated despite the availability of the vaccine, is becoming widespread worldwide and is, according to the World Health Organization (WHO), one of the top ten health threats in the world [19]. For several years, we observed the re-emergence of diseases that were close to being eradicated (measles…) [3], and recently, the Coronavirus disease (COVID-19) crisis illustrated difficulties in the fight against emerging diseases [20].

Thus, a better understanding of these new challenges for public health organizations is necessary to implement programs of prevention [15,21]. Developing prevention regarding identified risks responds now to a twofold problem: improving the population’s state of health (good quality of life, high number of years of life in good health), and seeking a better rationalization of care resources (pharmaceutical or technological) to keep the risk-benefit balance.

In France, the scourge of bacterial resistance, polymedication and vaccine hesitancy is more important than in other European countries. The consumption of antibiotics is 30% higher than the average European rate [22]. Excessive polymedication (HR 1.83 [1.28–2.62]) is detected as a predictor of mortality [23]. In addition, France has significantly greater disparities than the rest of Europe in terms of health (morbidity, mortality, perceived health and functional health). The main determinants of these inequalities are, in particular, working conditions, which are strongly linked to the socio-professional category [24]. This statement suggests the high relevance of selective or even indicated preventive actions [25]. In line with Antonovsky’s salutogenésis [26], working on knowledge, attitudes, and practices (KAP) in a specific professional setting, should make it possible to better develop appropriate informative and preventive actions, in order to create health, improving workers’ self-resources.

Among the overall population, permanent employees of the French public service represent an interesting socio-professional category to study for several reasons. Firstly, these public agents evolve in a specific work context, with a job guarantee, and relatively strong stability in their professional environment. Secondly, several socio-professional categories are represented [27]. Thirdly, this population has few occupational physicians, and some organizations must fill this gap in the prevention network. Finally, the management of health expenses for public service workers, is unique in the French public health system, with accounting management delegated to the mutualist system, and continuity of this management after their retirement. Thus, mutualist organizations have become important actors in preventive actions by a delegation from the public authorities as specified in the last Public Service Occupational Health Plan [28].

This retrospective study aims to analyze KAP related to medication, antibiotics, and vaccination among the public service population, from a mutualist union database. The results will allow for the establishment of the basis for implementing selective preventive actions for targeting sub-populations with specific needs of environmental resources.

## 2. Materials and Methods

### 2.1. Study Design

This study was designed as an ancillary study, from a cross-sectional anonymous online questionnaire-based survey that was conducted from 10 September to 7 October 2018. This research was performed in accordance with the Checklist for Reporting Of Survey Studies (CROSS) (Supplementary Section S1) [29].

The main study was run and promoted by UROPS (*Union Prévention Santé pour la Fonction publique*), a public organization unifying 11 mutual insurance companies, and managing 1.7 million insurees (The Morice Law of 1947 [30]). Under the Public Service Compulsory Scheme, the UROPS organization is involved in the deployment of preventive healthcare. Each year, UROPS conducts a survey of the KAP of its beneficiaries on various public health topics in order to initiate specific preventive actions (risk identification, information, formation…). In 2018, the questionnaire “barometer” was specifically developed by the UROPS members to analyze KAP about medication, antibiotics and vaccination, three primary care general public health concerns. It also aimed to provide an overview of the situation regarding contact persons and information, in case of health needs. For the “barometer 2018” princeps study, cohort management was entirely online, including invitations, registration, and data collection. The largest share of participants was recruited through an email invitation sent by UROPS to its insurees.

The ancillary study consisted of a retrospective analysis of the “barometer 2018”, based on a selection of the questions focused on participants’ KAP related to medications including antibiotics and hypnotics, and vaccination.

### 2.2. Selection of Respondents

To be included in the questioned-based survey, the participants had to be (i) at least 18 years old, (ii) public service agents or have been public service agents, (iii) insured by the insurance union for public service workers UROPS, (iv) registered on the website from the public insurance [31] and have agreed to be contacted via their personal email address on this channel, (v) fluent in the French language, (vi) agree to fulfill the online questionnaire, (vii) be able to use the internet via PC or mobile device. The exclusion criteria were third-party beneficiaries of UROPS members and non-permanent employees.

### 2.3. Ethics

Our protocol and study design were approved by ethics and regulatory agencies and were implemented in accordance with provisions of the Declaration of Helsinki. The appropriate Committee (Local Research Ethics Committee, Rouen, France) approved the protocol on 22 August 2022 (Ref E2022-38).

### 2.4. Measurement Tool

The participant’s KAP related to antibiotics, polymedication and vaccination were assessed by a selection of 33 questions extracted without adaptation from the initial survey composed of four sections (Supplementary Section S2).

The first section referred to patient characteristics with 13 questions, especially gender, age by 10 years, marital status, number and age of children living in the household, current work situation, the highest degree of obtained diploma (Level 1: Level of education below “Baccalaureate” (French high school diploma) degree, Level 2: Level of education greater than or equal to “Baccalaureate” degree and less than or equal to “Baccalaureate” degree plus 2 years, Level 3: Level of study higher than “Baccalaureate” degree plus 2 years, the last statutory category, the size of the municipality of residence, the region of residence, the presence of chronic illness, a disability or a health problem for at least 6 months requiring regular care or treatment, and the health insurance coverage.

The second section concerned knowledge and attitudes regarding antibiotics with 5 questions. The first question referred to a series of 4 statements to which the respondent is asked to confirm or not, or refrain from answering (yes or no or don’t know). The following three questions referred to their attitudes regarding antibiotics. The last question consisted in ranking the information carriers (including health and non-health professionals), depending on the confidence levels.

The third section collected attitudes regarding medications with 10 questions, especially concerning the number of medications per day, the treatment consumption for sleeping or treating stress and/or anxiety, and their feelings about their treatments.

The last section of the questionnaire dealt with knowledge and practices concerning vaccination with 5 questions, among them a series of 4 statements to which the respondent was asked to confirm or not, or refrain from answering (yes or no or don’t know). The other questions referred to attitudes and levels of access to vaccination. A question consisted in ranking the information carriers (including health and non-health professionals), depending on the confidence levels.

### 2.5. Statistical Analysis

From the collected responses, new variables were constituted:

- The levels of knowledge related to antibiotics or vaccination were evaluated post-collection using the responses of the 4 statements; “low” when zero or 1 right answer was provided, “moderate” for 2 or 3 right answers and “high” for 4 right answers;
- Polymedication has been defined as taking at least 3 medications per day;
- Antibiotic misuse was defined as (i) self-interruption of the prescription duration, or (ii) taking non-prescribed antibiotics for himself, his children or relatives. The polymedicated respondents were identified as respondents with at least 3 medications per day;
- A proactive behavior was identified among respondents when: (i) self-evaluation by the respondent of the need for antibiotics and asking the doctor for a prescription, or (ii) they had already discussed their medication with their physician/pharmacist on their own initiative, or (iii) if they were taking medication for better sleep, they had already tried non-medicinal methods, or (iv) if they were taking medication for stress or anxiety, they had already tried non-medicinal methods;
- Vaccinophobia was defined by the combination of four kinds of behavior: The vaccinophobic respondents were identified as respondents (i) who are not up to date with their vaccines, and (ii) not interested in receiving additional information on vaccines or vaccination, (iii) would not be vaccinated regardless of the conditions of access to vaccines, and (iv) would not be vaccinated regardless of the cost of vaccines.

Categorical data were presented as frequencies and percentages. Bivariate statistical comparisons were performed with Pearson’s χ^2^ test for categorical data. The proportions of respondents per French metropolitan region were compared with the data from the French population census (obtained at the end of 2021 from the national institute of demographic studies). A non-parametric statistical test was used to compare the non-independent proportions of respondents in the sample study and inhabitants per region. A probability value, *p*, of less than 0.05 was considered significant.

A Multiple Correspondence Analysis (MCA) was used to summarize the information contained in a large number of variables to facilitate the interpretation of the existing correlations between these different variables and to determine a posteriori sub-group of interest for descriptive statistics. The variables considered in the MCA were: gender, age, marital status, level of education, last professional category, region of residence, presence or absence of chronic disease and occupational status (worker or retired).

Binary logistic regressions were performed on the retained subgroups from the MCA to evaluate the determinants of the misuse of antibiotics, the polymedication, daily anxiolytic or hypnotic treatments and vaccinophobia. Odds ratio with a 95% confidence interval and *p*-values were provided. Covariates were gender, age, education levels, the presence of at least one dependent child in the household, occupational status, the presence of at least one chronic disease, the confidence in physician or pharmacist in antibiotics and vaccines, and the level of knowledge in antibiotics and vaccines.

Statistical analyses were performed with R using the “ade4”, “FactoMineR”, and “factoextra” packages from the R Project for Statistical Computing (version 4.1.1. 2021-08-10) (R Foundation for Statistical Computing, Vienna, Austria). A *p*-value below 0.05 was considered statistically significant.

## 3. Results

### 3.1. Characteristics of Respondents and Sample Size

**Figure 1:**
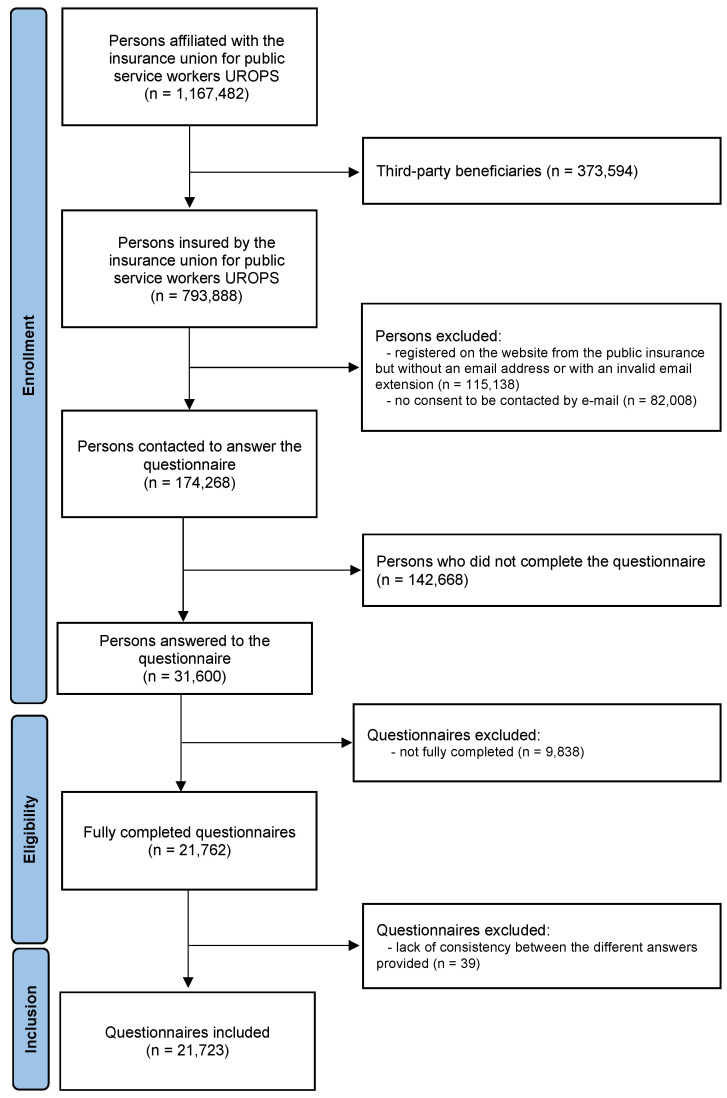
Flowchart of the study. UROPS: Union Prévention Santé pour la Fonction publique.

All respondents were invited to participate in the barometer, i.e., 174,268 people. Among those, 31,600 (18%) insurees participated in this survey. Of those who completed the online questionnaires, 21,762 were considered eligible, with all questions answered, and 9838 were excluded because participants dropped out during the survey. Of the 21,762 eligible questionnaires, 21,723 were included, with all completed questions, and 39 were rejected because they presented a lack of consistency between the different answers provided (Figure 1). The targeted population concerned the 174,268 persons insured by the UROPS, and 31 600 persons answered the questionnaires, thus the response rate of the final sample was 18.13%.

The final sample comprised 21,723 included respondents of whom 71.3% completed the survey using a laptop (15,482) and 29.7% a cell phone (6241). Among the included respondents, 47.0% were under 60 years old (10,206) and 53.0% were over 60 years old (11,517) (Supplementary Section S4: Figures S1 and S2). The age ranged from 18 to 80 years and older. The gender distribution was 41.1% males (8938) and 58.9% females (12,785). The majority of the included respondents were married (12,785, 58.9%). Nearly a quarter of the included respondents had at least one child living in the household (5348, 24.6%). Included respondents over 60 years old were 34.8% to have their “Baccalaureate” degree versus 52.9% of the included respondents under 60 years old. Regarding occupational status, included respondents were workers (11,014, 50.7%) or retired (10,709, 49.3%) in the same proportion. Moreover, 31% of included respondents (6734) declared having a chronic disease, a disability or a health problem. In the following results, the short-term “respondents” is used to design the “included respondents”.

### 3.2. Distribution of Respondents According to the Occupational Status

**Figure 2:**
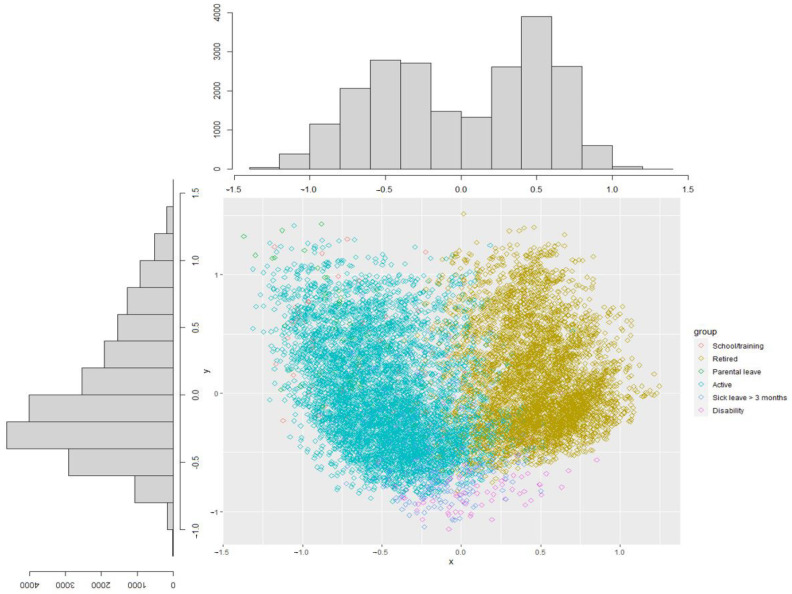
Projection of the distribution of the modalities of occupational status on the respondents’ positions on the two first factorial axis.

The projection of the sociodemographic variables on the MCA results showed that occupational status was the least overlapping group of modalities (Supplementary Section S3: Figures S5 and S6). The projection highlighted a marked vertical dichotomy (Figure 2); the workers were on the left and the retired were on the right. The worker group corresponds to active persons, parental leave, school training, and those who would stick to leaves > 3 months, whereas the retired group corresponds to persons retired from public service whatever their age and cause. These two groups based on occupational status were considered the most dichotomous of the sample respondents. The results of the analysis did not identify other socio-demographic variables that were sufficiently contributive to manage the statistical analysis of the survey questions to another specific subgroup analysis.

### 3.3. Socio-Demographic Characteristics According to the Occupational Status

The socio-demographic characteristics of the sample are displayed in Table 1. Retired were significantly more affected by chronic disease or disability or health problems than workers (*p* < 0.0001). They mainly suffered from heart, artery, vein, stroke disease and metabolic diseases.

### 3.4. Knowledge, Attitudes and Practices Related to Antibiotics, Medication and Vaccination According to the Occupational Status

Table 2 presents the results concerning KAP related to antibiotics, medication and vaccination among public service workers and retired.

Regarding antibiotics, 31.4% of working people (3456) versus 17.9% of retired (1918) had a high level of knowledge. The main part of respondents (73.90% of workers (8143) and 68.90% of retired (7378)) knew that antibiotics were ineffective against viruses. The question with the most incorrect responses was that antibiotics, in general, do not lead to faster healing (43.3% of workers (4766) and 28.70% of retired (3076)). The majority of workers (6747, 61.3%) and retired (7685, 71.8%) had good use of antibiotics.

Among retired, 20.4% took anxiolytics in the last few months (2188), 18.4% took hypnotics in the last few months (1974), 76.3% took medication daily (8172) and 24.4% were polymedicated (more than 3 medications per day) (2618), while among workers, 20.3% took anxiolytics in the last few months (2241), 18.4% took hypnotics in the last few months (1974), 49.0% took medication daily (5400) and 8.7% were polymedicated (956). A majority of workers (9450, 85.8%) and retired (8741, 81.6%) were described as proactive. They actively self-evaluated their antibiotic needs and demanded them (79.0% of workers (8703) and 70.4% of retired (70.4%)).

Concerning vaccination, 33.8% of workers (3718) and 31.6% of retired (3389) had a high level of knowledge. The question with the most incorrect answers concerned the fact that vaccines cause side effects. More than half of the respondents, whether they were working or retired, were convinced of this. There were few vaccinophobics among workers (93, 0.8%) and retired (186, 1.7%). Among respondents, 10.5% of workers (1155) and 15.3% of retired (1643) declared not to be updated with vaccination. More than half of the workers (7891, 71.6%) and retired (8307, 75.0%) stated that they were not interested in having information about the vaccine schedule and/or the composition of vaccines and/or the current state of scientific knowledge about vaccines and/or the recommended vaccines for foreign travel. Even if vaccination was possible at the workplace and/or in a pharmacy, workers (3239, 29.4%) and retired (3785, 35.3%) would not change their intention to get vaccinated. The reduction in the cost or the free vaccine would not change the intention of workers (4305, 39.1%) as retired (4560, 42.6%) to get vaccinated.

### 3.5. Determinants of Risky Behaviors and Practices with Public Service Population in France

#### 3.5.1. Determinants of the Polymedication

**Figure 3:**
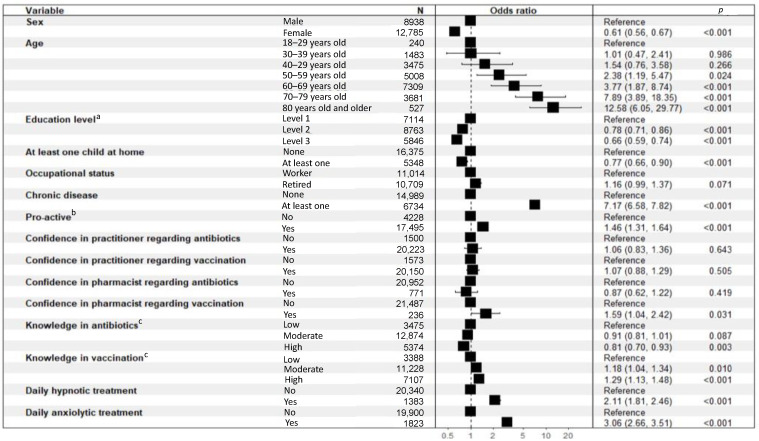
Binary logistic regressions performed to evaluate the determinants of polymedication. ^a^ Level 1: Level of education below “Baccalaureate” (French high school diploma) degree, Level 2: Level of education greater than or equal to “Baccalaureate” degree and less than or equal to “Baccalaureate” degree plus 2 years, Level 3: Level of study higher than “Baccalaureate” degree plus 2 years; ^b^ Proactive: ((asks physician for antibiotics) OR (if taking meds, has already discussed it with physician/pharmacist on own initiative) OR (if med for better sleep, has already tried non-medication methods) OR (if medication for anxiety, has already tried non-medication methods)), ^c^ Low: 0 or 1 right answer, Moderate: 2 or 3 right answers, High: 4 right answers.

The results of the binary logistic regressions performed to evaluate the determinants of the polymedication are presented in Figure 3. Polymedication was associated with higher proportions of females, over 40 years old (OR = 1.54 (0.76–3.58)), having at least one chronic disease (OR = 7.17 (6.58–7.82); *p* < 0.001), proactive (OR = 1.46 (1.31–1.64); *p* < 0.001), confident in the pharmacist for vaccination (OR = 1.59 (1.04–2.42); *p* = 0.031), using daily hypnotic (OR = 2.11 (1.81–2.46); *p* < 0.001) or anxiolytic (OR = 3.06 (2.66–3.51); *p* < 0.001) treatment. Those with a level of education equal to or higher than a “Baccalaureate” degree (OR = 0.78 (0.71–0.86); *p* < 0.001), having at least one child at home (OR = 0.77 (0.66–0.90); *p* < 0.001), having a high level of antibiotic knowledge (OR = 0.81 (0.70–0.93); *p* = 0.003) were less polymedicated.

#### 3.5.2. Determinants of the Daily Use of Anxiolytic or Hypnotic Treatments

**Figure 4:**
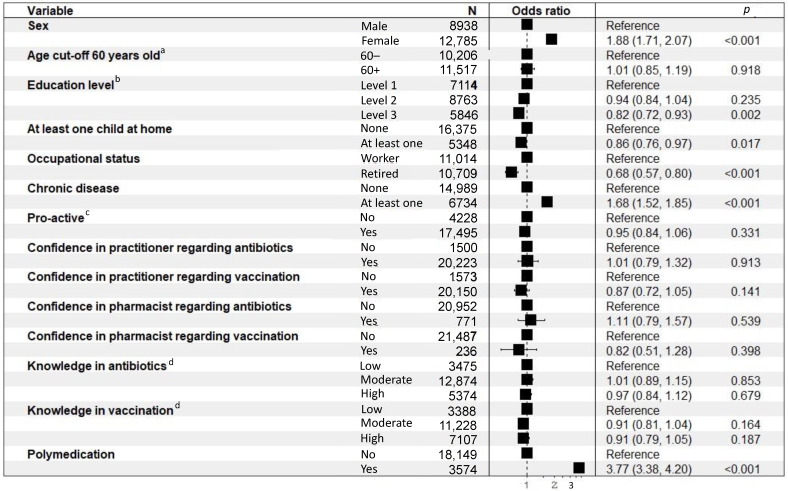
Binary logistic regressions performed to evaluate the determinants of daily anxiolytic or hypnotic treatments. ^a^ The age cut-off was determined at 60 years old to have homogenous age range; ^b^ Level 1: Level of education below “Baccalaureate” (French high school diploma) degree, Level 2: Level of education greater than or equal to “Baccalaureate” degree and less than or equal to “Baccalaureate” degree plus 2 years, Level 3: Level of study higher than “Baccalaureate” degree plus 2 years; ^c^ Proactive: ((asks physician for antibiotics) OR (if taking meds, has already discussed it with physician/pharmacist on own initiative) OR (if med for better sleep, has already tried non-medication methods) OR (if medication for anxiety, has already tried non-medication methods)), ^d^ Low: 0 or 1 right answer, Moderate: 2 or 3 right answers, High: 4 right answers.

The results of the binary logistic regressions performed to evaluate the determinants of anxiolytic or hypnotic treatments are presented in Figure 4. Female (OR = 1.89 (1.72–2.09); *p* < 0.001), over 30 years old (OR > 2.14 (1.15–4.45); *p* ≤ 0.026), having at least one chronic disease (OR = 1.67 (1.51–1.84); *p* < 0.001), polymedicated (OR = 3.73 (3.34–4.17); *p* < 0.001) used more daily anxiolytic or hypnotic treatments. People with a level of study equal or superior to the “Baccalaureate” degree plus 2 years (OR = 0.82 (0.72–0.93); *p* = 0.002), having at least one child at home (OR = 0.86 (0.75–0.97); *p* = 0.017), retired (OR = 0.68 (0.57–0.80); *p* < 0.001) used less daily anxiolytic or hypnotic treatments.

#### 3.5.3. Determinants of the Misuse of Antibiotics

**Figure 5:**
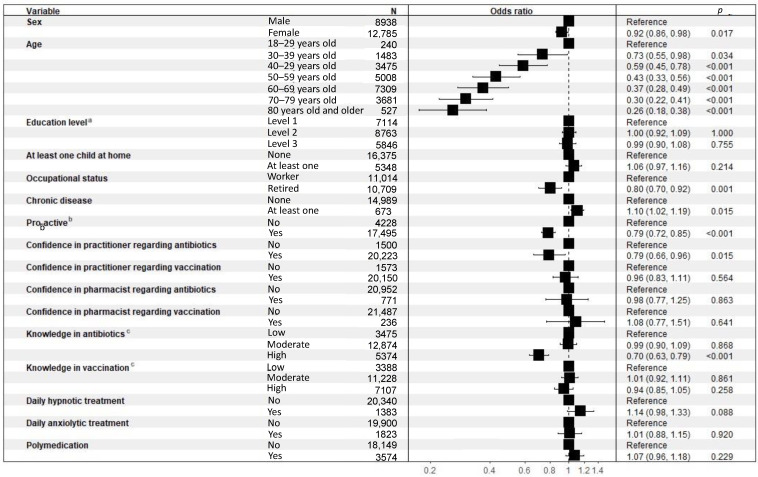
Binary logistic regressions performed to evaluate the determinants of the misuse of antibiotics. ^a^ Level 1: Level of education below “Baccalaureate” (French high school diploma) degree, Level 2: Level of education greater than or equal to “Baccalaureate” degree and less than or equal to “Baccalaureate” degree plus 2 years, Level 3: Level of study higher than “Baccalaureate” degree plus 2 years; ^b^ Proactive: ((asks physician for antibiotics) OR (if taking meds, has already discussed it with physician/pharmacist on own initiative) OR (if med for better sleep, has already tried non-medication methods) OR (if medication for anxiety, has already tried non-medication methods)), ^c^ Low: 0 or 1 right answer, Moderate: 2 or 3 right answers, High: 4 right answers.

The results of the binary logistic regressions performed to evaluate the determinants of the misuse of antibiotics are presented in Figure 5. Respondents over 40 years old (OR = 0.43 (0.33–0.56); *p* < 0.001), retired (OR = 0.8 (0.71–0.91); *p* = 0.001), proactive (OR = 0.75 (0.69–0.81); *p* < 0.001), confident in practitioner about antibiotics (OR = 0.67 (0.57–0.79); *p* < 0.001), and having high level of knowledge about antibiotics (OR = 0.62 (0.56–0.69); *p* < 0.001), practiced less antibiotic misuse. Respondents with at least one child at home practiced more antibiotic misuse (OR = 1.11 (1.02–1.21); *p* = 0.015).

#### 3.5.4. Determinants of Vaccinophobia

**Figure 6:**
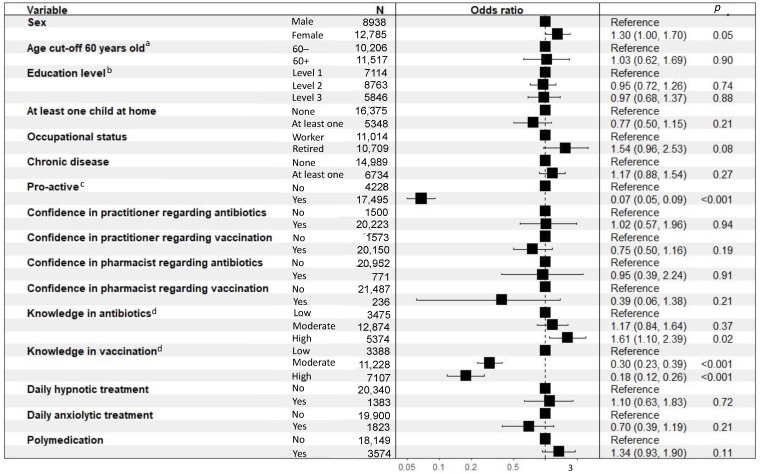
Binary logistic regressions performed to evaluate the determinants of vaccinophobia. ^a^ The age cut-off was determined at 60 years old to have homogenous age range; ^b^ Level 1: Level of education below “Baccalaureate” (French high school diploma) degree, Level 2: Level of education greater than or equal to “Baccalaureate” degree and less than or equal to “Baccalaureate” degree plus 2 years, Level 3: Level of study higher than “Baccalaureate” degree plus 2 years; ^c^ Proactive: ((asks physician for antibiotics) OR (if taking meds, has already discussed it with physician/pharmacist on own initiative) OR (if med for better sleep, has already tried non-medication methods) OR (if medication for anxiety, has already tried non-medication methods)), ^d^ Low: 0 or 1 right answer, Moderate: 2 or 3 right answers, High: 4 right answers.

The results of the binary logistic regressions performed to evaluate the determinants of vaccinophobia are presented in Figure 6. Females (OR = 1.30 (1.00–1.70); *p* = 0.05), retired (OR = 1.54 (0.96–2.53); *p* = 0.08), having a high level of knowledge in antibiotics (OR = 1.61 (1.10–2.39); *p* = 0.02) were more vaccinophobic. Proactive respondents (OR = 0.07 (0.05–0.09); *p* < 0.001), having a moderate level (OR = 0.30 (0.23–0.39); *p* < 0.001) or high level (OR = 0.18 (0.12–0.26); *p* < 0.001) of knowledge in vaccination were less vaccinophobic. The confidence in practitioner (OR = 0.75 (0.50–1.16); *p* = 0.19) or pharmacist (OR = 0.39 (0.06–1.38); *p* = 0.21) concerning vaccination presented a dispersion of variables and were not significantly associated with less vaccinophobia.

## 4. Discussion

To the best of our knowledge, this is the first study analyzing KAP related to medication, antibiotics, and vaccination among the public service population. Understanding the barriers to reducing the consumption of anxiolytics and hypnotics, decreasing polymedication, promoting adequate antibiotic use and minor vaccination hesitancy is a prerequisite for implementing efficient preventive actions. To meet this challenge, this study proposed an original design because complex variables were created a posteriori, based on the association of attitudes, collected in different questions. Thus, to analyze behavior towards antibiotics and polymedication, antibiotic misuse and proactivity were studied, while behaviors linked to vaccination were analyzed through vaccinophobia.

### 4.1. Key Points Concerning the Population

This study on a targeted population has several strengths. First, a wide proportion of the adult population was included because the age ranged from 18 to 80 years of and above (from the legal working age to death (taking into account retirees)). Secondly, the public service population represents the main part of socioprofessionnal category with a wide variety of public occupations (clerks, customs officers, librarians, police officers, state architects, magistrates, port officers, prison staff, etc.). Thirdly, the sample studied was representative of the territorial repartition. Fourth, the population studied included a large proportion of elderly people, at risk of illness, or already suffering from chronic diseases that are associated with increased use of healthcare services. Fourth, the response rate of the final sample was consistent with that observed for the e-mail surveys and the size of the sample studied allows logistic regressions to be performed. Confidence intervals are restricted, which attests to a sufficient sample size and a factor of relevance to the results.

Compared with other populations, the public service population was particularly specific because several socio-demographic variables, usually known to impact KAP demonstrated no significant effect in our study. Sex, education level, marital status and the fact of suffering from a chronic disease that have been identified as determinants in other studies [32,33,34,35] did not statistically modify the KAP in this public service population. However, the occupational status, which is mainly linked to age but not only, allowed for the dichotomization of the sample respondents between workers and retired. Thus, the study tried to determine if occupational status could be considered a determinant of KAP related to medication, antibiotics, and vaccination.

According to the multivariate analysis, workers had different KAP than retired, without shared determinants in the three health domains studied (polymedication, antibiotics and vaccination). Thus, these themes should be studied independently. Anxiolytic and hypnotic treatments were associated with a higher risk of polymedication, growing with age and not associated with chronic disease status. This provides larger data than classically studies focusing on anxiolytic-hypnotic polypharmacy [36,37,38].

### 4.2. Key Points Concerning the Anxiolytics and Hypnotics

Hypnotic and anxiolytic consumption must be controlled because these drugs can lead to undesirable effects as well as health consequences and costs. First, it has been shown that the use of psychotropic drugs in the workplace can reduce performance and cause accidents [39]. Second, these drugs are highly involved in voluntary self-poisoning [40]. Due to the fact that workplace suicide has become an urgent social concern internationally with rising numbers of employees choosing to kill themselves in the face of extreme pressures at work. In France, suicides have affected a wide range of public companies (telecommunications giant as *France Télécom*, French postal services, electricity and gas suppliers, police forces and research centers) [41]. It has been shown that working conditions play a role in psychotropic drug use [42,43,44,45,46], even if the fatal outcome is not systematic. Particularly, in the French working population, several occupational factors, including a large set of psychosocial work factors were identified such as psychological demands, low social support and hiding emotions were associated with psychotropic drug use [47].

Considering the main results of this study, the use of psychotropic drugs (anxiolytics (20.4%) and hypnotics (21.4%)) by the public service population was similar to that of the general French population (19.4%) [48]. Classical risk factors of psychotropic consumption such as female gender and older age were identified, as in previous studies [47,48]. The relevance of selected prevention relative to these concerns in comparison to a classical approach is not obvious.

### 4.3. Key Points Concerning the Polymedication

Regarding polymedication, a study based on data from 17 European countries and Israel reported that the prevalence of polymedication in adults aged 65 years ranged from 26.3 to 39.9% [49]. In our study, the results were similar as 24.4% of retired people were polymedicated. Polymedication leads to an increased risk of adverse events and drug interactions [50]. In addition, polymedication was associated with an increased risk of medication misuse, longer hospitalization and mortality [23,50]. It was demonstrated that a better understanding of patients’ perceptions of their medications could help reduce the burden of polymedication. Patients’ beliefs and attitudes towards medications impacted the propensity to accept deprescription. The first International Group for Reducing Inappropriate Medication Use and Polypharmacy proposed 10 actions to prevent polymedication [51]. The authors propose a return to the original concept of evidence-based medicine that incorporated patient preference, context, clinical judgment, and scientific data. A study of Swiss elderly patients concluded that 97% of patients with polymedication were satisfied with their medications, but 16% felt that their medications were a burden [52]. A previous study conducted in France concluded that over 50% of adults living in the Paris metropolitan area were self-medicated in the past four weeks [53]. In our study, 83.7% of respondents were proactive. The majority of respondents (79.0% of workers (8703) and 70.4% of retired (70.4%)) actively self-evaluated their antibiotic needs and demanded them. Special attention must be provided to these individuals. In order to improve their knowledge and behavior with regard to medication, targeted prevention, education and health promotion actions must be implemented as the first International Group for Reducing Inappropriate Medication Use and Polypharmacy proposed [51]. For them, a reconceptualization of the medical care framework is necessary to better serve patients with multimorbidity, which involves changes in medical education, quality measures, and policy.

### 4.4. Key Points Concerning the Antibiotics

According to Eurobarometer data, 39% of French took antibiotics at least once in 2016 [54]. Public knowledge, attitudes, and beliefs about antibiotics were strong determinants of antibiotic misuse [55]. The main part of the French general population (84%) knew that the unnecessary use of antibiotics makes them become ineffective. However, their knowledge about antibiotics needs to be improved as 41% did not know that antibiotics were ineffective against viruses, and 33% did not know that antibiotics had no effect against colds and influenza [54]. In our study, the level of knowledge of the public service population studied was higher than the general French population because 71.5% of the respondents (73.9% of workers and 68.9% of retired) knew that antibiotics were ineffective against viruses and 91.4% (92.9% of workers and 89.8% of retired) knew that unnecessary use of antibiotics makes them become ineffective. Despite this high level of knowledge about antibiotics, 28% of respondents misuse them. They took non-prescribed antibiotics for himself or for his children or for his relatives (21%) or stopped taking antibiotics before the end of the prescription (12.1%). The practice of self-medication was facilitated by leftover antibiotics from previous prescriptions when the patient has not complied with the treatment or the number of antibiotics prescribed had exceeded the duration of the treatment [3]. According to Eurobarometer, 2 percent of Europeans used antibiotics left over from previous courses [54]. In the United Kingdom, a survey of 6983 households showed that 19% of respondents had a remaining prescription. Prescriptions older than six days accounted for 61% of remaining medications, while prescriptions less than three days old accounted for 6% of remaining medications [56].

This study provided expected results regarding antibiotic misuse. Respondents over 40 years old or retired, proactive, confident in practitioner about antibiotics, and having a high level of knowledge about antibiotics, practiced less antibiotic misuse. These results are in agreement with those of Guo et al. (2022) who observed that adults over 50 years old with poor knowledge of antibiotic use had a 3× increased odds of inappropriate antibiotic use in adults aged ≥ 50 years [55].

Moreover, as misuse of antibiotics is the first driver of antibiotic resistance [57], it appears necessary to raise public awareness about antibiotic resistance and the correct use of antibiotics. Public awareness must be accompanied by emotional or material incentives, a supportive social structure, and a strong regulatory environment in order to achieve real behavioral change [57]. In 2015, the WHO published a global action plan on antibiotic resistance and advised member states to implement their own action plan [58]. The main objective in 2015 was to improve awareness and understanding of antimicrobial resistance through effective communication, education and training. To improve the impact of such global plans, healthcare professionals can help prevent the development of antibiotic resistance [59] by (i) adopting antibiotic stewardship programs, (ii) improving diagnosis, monitoring, and prescribing practices, and (iii) optimizing treatment regimens and preventing the transmission of infections [60]. Policies, initiatives, and investments in new agents also have a role to play [60]. In addition, patients in healthcare facilities are at increased risk of infection with common pathogens, which are a factor in antibiotic resistance. To avoid this, infection prevention and control measures must be implemented in healthcare facilities and the broader community to reduce the spread of pathogens, particularly resistant agents. One tool is vaccination, which can act directly and indirectly to reduce the prevalence of the resistant pathogen and the use of antibiotics [61]. Another is good hand hygiene practice that is essential to fight against nosocomial infection [59].

### 4.5. Key Points Concerning the Vaccinophobia

As shown in the multivariate analysis, the proportions of females and retired respondents, having a high level of knowledge of antibiotics, were more vaccinophobic compared with others. On the contrary, proportions of proactive respondents, having a moderate level or high level of knowledge of vaccination were less vaccinophobic than others. Only 0.8% of public service workers and 1.7% of retired were defined as vaccinophobics. Thus, these results suggest that vaccination awareness and prevention activities should not be a priority for public service workers and retired. However, these results date from before the COVID-19 health crisis and should be taken with caution. Vaccinophobia is a public health issue that re-emerged in 2019. An online study conducted in France qualified 35% of respondents as “COVID-19 vaccine hesitant” [62]. COVID-19 anxiety and health-related fears were linked to higher vaccine acceptance, whereas fear of social and economic impact indicated the reverse direction [63]. Health literacy should be considered to decrease vaccinophobia [64]. Greater scores in health literacy and detection of fake news were associated with the intention to get vaccinated [65].

### 4.6. Key Points Concerning Confidence in Trusting Information Source

In our study, the respondents had to classify in decreasing order the most trustworthy carriers of information related to antibiotics and vaccination. In the first row, there was a general practitioner/pediatrician (93.1%) for antibiotic information and (92.8%) for vaccination information. In the second row, there was a pharmacist (3.5%) for antibiotic information and a public authority website (2.5%) for vaccination information. In the third row, there was a public authority website (1.5%) for antibiotic information and a pharmacist (1.5%) for vaccination information. These results were concordant with the results of the Eurobarometer (2016) that permitted multiple choices [54]. Regarding finding information about antibiotics, respondents saw medical professionals as the most trustworthy carriers of information. Doctors were identified by 84% of respondents as an important source of information, while 37% would use a pharmacy to obtain trustworthy information, 19% would obtain information from a hospital, and 15% would visit an official health-related website. In terms of looking for information on vaccination, it is interesting to have this data from 2018, shortly before the COVID-19 crisis. They may be able to better understand the evolution of vaccine information management and the acceptance process for the delegation of health tasks. For example, in France, pharmacists played a central role in the fight against COVID-19, their functions having been expanded because they participated in the detection of the disease, but also by dispensing the act of vaccination.

### 4.7. Limitations

This study has several limitations. First of all, this study is an ancillary study, the sample size was not determined preliminarily. However, the sample size matches the quality criteria for survey research and reports [66]. Second, in this “French mutual insurees” centered study, the respondents corresponded to a very specific population, in a specific health system regarding health costs management. Thus, despite territorial representativity, the results and conclusions cannot be generalized to a large population without precautions. Third, a selection bias exists because only those who registered on the public insurance website [31] and agreed to be contacted via their personal email address on this channel were contacted to participate in this study. It is, therefore, possible that these people had the facilities to use the internet and can therefore access information more easily. Fourth, the results were based on self-reported data. We were not able to cross-reference the questionnaire responses with actual practices of antibiotic use, medication, and vaccination. Social desirability bias may also have impacted the credibility of our results. Fifth, the survey was not designed to analyze the impact of professional categorization and geographical repartition. Sixth, awareness of the public health impact of antibiotic resistance and the level of understanding of antibiotics were only analyzed indirectly by studying the misuse of antibiotics [57]. Thus, given the lack of publications on the very specific population of French public agents, this study provides a better understanding, but further studies are needed with a specific design.

## 5. Conclusions

The analysis of KAP related to medications, antibiotics and vaccination among the public service population revealed a dichotomization of respondents according to their professional status. Public service workers have different KAP than retired, without shared determinants in the 3 health domains studied. Nevertheless, public service workers and retired shared major common characteristics. They were highly exposed to polymedication, and hypnotic overtake, and one-third of them misuse antibiotics despite a good level of knowledge. There did not seem to be any concern for vaccinophobia before the COVID-19 crisis. Thus, selective and indicated prevention approaches should be implemented. Actions related to antibiotics and polymedication should be a priority, before vaccination. In this study, the main part of respondents were proactive individuals, using multiple sources of trustworthy information, and appeared to be ready for the delegation of health tasks. Mutual organizations should be involved as relevant actors. Studying their insurees longitudinally could be interesting to highlight the impact of selective prevention on behaviors, through trusted health professionals (general practitioners, pharmacists…).

## Figures and Tables

**Table 1:** Socio-demographic characteristics and chronic illness categories of the respondents according to occupational status. The results are expressed as n (%). n: number of respondents, %: percentage.

Variable	All (N = 21,723)	Workers (N = 11,014)	Retired (N = 10,709)	*p*-Value
**Gender**				<0.0001
male	8938 (41.1)	3887 (35.3)	5051 (47.2)	
female	12,785 (58.9)	7127 (64.7)	5658 (52.8)	
**Age range**				<0.0001
18–29 years	240 (1.1)	240 (2.2)	0 (0.0)	
30–39 years	1483 (6.8)	1481 (13.4)	2 (0.0)	
40–49 years	3475 (16.0)	3470 (31.5)	5 (0.0)	
50–59 years	5008 (23.1)	4620 (41.9)	388 (3.6)	
60–69 years	7309 (33.6)	1185 (10.8)	6124 (57.2)	
70–79 years	3681 (16.9)	11 (0.1)	3670 (34.3)	
≥80 years	527 (2.4)	7 (0.1)	520 (4.9)	
**Marital status**				<0.0001
Single	2592 (11.9)	1935 (17.6)	657 (6.1)	
Divorced	2457 (11.3)	1249 (11.3)	1208 (11.3)	
Married	12,794 (58.9)	5399 (49.0)	7395 (69.1)	
Civil union	1240 (5.7)	1096 (10.0)	144 (1.3)	
Common-law	1524 (7.0)	1162 (10.6)	362 (3.4)	
Widowed	1116 (5.1)	173 (1.6)	943 (8.8)	
**Children living in the household**				<0.0001
0	16,375 (75.4)	6050 (54.9)	10,325 (96.4)	
>1	5348 (24.6)	4964 (45.1)	384 (3.6)	
**Level of study**				<0.0001
Below “Baccalaureate” degree	7114 (32.7)	2224 (20.2)	4890 (45.7)	
Greater than or equal to “Baccalaureate” degree and less than or equal to “Baccalaureate” degree plus 2 years	8763 (40.3)	5049 (45.8)	3714 (34.7)	
Higher than “Baccalaureate” degree plus 2 years	5846 (26.9)	3741 (34.0)	2105 (19.7)	
**Chronic disease or disability or health problem**				<0.0001
Yes	6734 (31.0)	2942 (26.7)	3792 (35.4)	
No	14,989 (69.0)	8072 (73.3)	6917 (64.8)	
**Type of chronic disease or disability or health problem ^1^**				
Respiratory problems	1301 (6.0)	591 (8.8)	710 (10.5)	
Heart, artery, vein or stroke disease	1825 (8.4)	475 (7.1)	1350 (20.0)	
Metabolic disease	2178 (10.0)	830 (12.3)	1348 (20.0)	
Tumors	869 (4.0)	294 (4.4)	575 (8.5)	
Mental illness	350 (1.6)	238 (3.5)	112 (1.7)	
Locomotor problems	1552 (7.1)	667 (9.9)	885 (13.1)	
Other	2194 (10.1)	1113 (16.5)	1081 (16.1)	

^1^ The percentage was calculated based on the number of people who reported having a chronic condition, disability, or health problem. A respondent could choose more than one answer. In bold, head of variable categories.

**Table 2:** Knowledge, attitudes and practices related to antibiotics, medication and vaccination among public service workers and retired.

Variable	All (N = 21,723)	Workers (N = 11,014)	Retired (N = 10,709)	*p*-Value
**ANTIBIOTICS**				
**Knowledge ^a^**				<0.0001
Low	3475 (16.0)	1451 (13.2)	2024 (18.9)	
Moderate	12,874 (59.3)	6107 (55.4)	6767 (63.2)	
High	5374 (24.7)	3456 (31.4)	1918 (17.9)	
Antibiotics ineffective against viruses	15,521 (71.5)	8143 (73.9)	7378 (68.9)	
Antibiotics effective against bacteria	16,116 (74.2)	8531 (77.5)	7585 (70.8)	
Taking antibiotics often can make them less effective	19,849 (91.4)	10,231 (92.90)	9618 (89.8)	
Antibiotics, in general, don’t make it possible to heal more quickly	7842 (36.1)	4766 (43.3)	3076 (28.70)	
**Antibiotics misuse**				<0.0001
Yes	6083 (28.0)	3659 (33.2)	2424 (22.6)	
No	15640 (72.0)	7355 (66.8)	8285 (77.4)	
Self-interruption of the antibiotics prescription duration	2628 (12.1)	1601 (14.5)	1027 (9.6)	
Take non-prescribed antibiotics for himself or for his children or for his relatives	4555 (21.0)	2809 (25.5)	1746 (16.3)	
**MEDICATION**				
**Anxiolytic intake in the last few months**				<0.0001
Yes	4429 (20.4)	2241 (20.3)	2188 (20.4)	
No	17,294 (79.6)	8773 (79.7)	8521 (79.6)	
**Hypnotic intake in the last few months**				<0.0001
Yes	4645 (21.4)	2671 (24.3)	1974 (18.4)	
No	17,078 (78.6)	8343 (75.7)	8735 (81.6)	
**Daily medication intake**				<0.0001
Yes (1 or more)	13,572 (62.5)	5400 (49.0)	8172 (76.3)	
No	8151 (37.5)	5614 (51.0)	2537 (23.7)	
**Polymedication ^b^**				<0.0001
Yes	3574 (16.5)	956 (8.7)	2618 (24.4)	
No	18,149 (83.5)	10,058 (91.3)	8091 (75.6)	
**Proactive behavior ^c^**				<0.0001
Yes	18,191 (83.7)	9450 (85.8)	8741 (81.6)	
No	3532 (16.3)	1564 (14.2)	1968 (18.4)	
Self-evaluation of its antibiotic needs and active request for it	16,242 (74.8)	8703 (79.0)	7539 (70.4)	
If medication intake, has already discussed it with physician/pharmacist on own initiative	4934 (22.7)	1712 (15.5)	3222 (30.1)	
If medication intake for sleep disorder, has already tried non-medication methods	2803 (12.9)	1511 (13.7)	1292 (12.1)	
If medication intake for anxiety, has already tried non-medication methods	3077 (14.2)	1859 (16.9)	1218 (11.4)	
**VACCINATION**				
**Knowledge**				<0.0001
Low	3388 (15.6)	1537 (14.0)	1851 (17.3)	
Moderate	11,228 (51.7)	5759 (52.3)	5469 (51.1)	
High	7107 (32.7)	3718 (33.8)	3389 (31.6)	
Useful to be vaccinated even against a disease that has disappeared	15,976 (73.5)	8696 (79.0)	7280 (68.0)	
Vaccination is better to develop its own immune defenses than to have the disease	15,342 (70.6)	7883 (71.6)	7459 (69.7)	
Vaccines are effective and useful	19,175 (88.3)	9680 (87.9)	9495 (88.7)	
Vaccines don’t cause serious side effects	9770 (45.0)	4872 (44.2)	4898 (45.7)	
**Vaccinophobia**				<0.0001
Yes	279 (1.3)	93 (0.8)	186 (1.7)	
No	21,444 (98.7)	10,921 (99.2)	10,523 (98.3)	
Not up to date with vaccinations	2798 (12.9)	1155 (10.5)	1643 (15.3)	
Not search for information about vaccine schedule, and/or composition of vaccines and/or current state of scientific knowledge about vaccines and/or recommended vaccines for foreign travel	15,928 (73.3)	7891 (71.6)	8307 (75.0)	
No intention to get vaccinated even if it is possible at the workplace and/or in a pharmacy	7024 (32.3)	3239 (29.4)	3785 (35.3)	
No intention to be vaccinated even if the cost is reduced and/or if the vaccine is free	8865 (40.8)	4305 (39.1)	4560 (42.6)	

The results are expressed as n (%). n: number of respondents, %: percentage. ^a^ Knowledge: Low (0 or 1 right answer), Moderate (2 or 3 right answers), High (4 right answers); ^b^ Polymedication: more than 3 medications by day; ^c^ Proactive: ((asks physician for antibiotics) OR (if taking meds, has already discussed it with physician/pharmacist on own initiative) OR (if med for better sleep, has already tried non-medication methods) OR (if medication for anxiety, has already tried non-medication methods)). In bold, head of variables categories.

## Data Availability

Data are available on request to the corresponding author.

## Supplementary Materials

The following supporting information can be downloaded at: https://www.mdpi.com/article/10.3390/ijerph192114044/s1, Section S1: Checklist for Reporting Of Survey Studies (CROSS); Section S2: Questionnaire on knowledge and practices concerning antibiotics, medication and vaccination; Section S3. Multiple Correspondence Analysis (MCA) results; Section S4. Repartition of the respondents according to age; Section S5. Knowledge, attitudes and practices related to polymedication, antibiotics, and vaccination among public service workers and retired.
