# Supplementary material for: Knowledge, Attitudes and Practices Related to Medication, Antibiotics, and Vaccination among Public Service Population: National Survey Conducted in France
Source: Int J Environ Res Public Health. 2022 Oct 28;19(21):14044. doi: 10.3390/ijerph192114044 (PMC9654407; doi:10.3390/ijerph192114044)
Supplement: Supplementary file 1 [file ijerph-19-14044-s001.zip › ijerph-1989703-supplementary.pdf]

S1. Checklist for Reporting Of Survey Studies (CROSS)

Section/topic	Item	Item description	Reported on page #
**Title and abstract**			
Title and abstract	1a	State the word "survey" along with a commonly used term in title or abstract to introduce the study's design.	1
	1b	Provide an informative summary in the abstract, covering background, objectives, methods, findings/results, interpretation/discussion, and conclusions.	1
**Introduction**			
Background	2	Provide a background about the rationale of study, what has been previously done, and why this survey is needed.	1-2
Purpose/aim	3	Identify specific purposes, aims, goals, or objectives of the study.	2
**Methods**			
Study design	4	Specify the study design in the methods section with a commonly used term (e.g., cross-sectional or longitudinal).	2-3
Data collection methods	5a	Describe the questionnaire (e.g., number of sections, number of questions, number and names of instruments used).	3-4
	5b	Describe all questionnaire instruments that were used in the survey to measure particular concepts. Report target population, reported validity and reliability information, scoring/classification procedure, and reference links (if any).	3-4
	5c	Provide information on pretesting of the questionnaire, if performed (in the article or in an online supplement). Report the method of pretesting, number of times questionnaire was pre-tested, number and demographics of participants used for pretesting, and the level of similarity of demographics between pre-testing participants and sample population.	NA
	5d	Questionnaire if possible, should be fully provided (in the article, or as appendices or as an online supplement).	S2
Sample characteristics	6a	Describe the study population (i.e., background, locations, eligibility criteria for participant inclusion in survey, exclusion criteria).	3
	6b	Describe the sampling techniques used (e.g., single stage or multistage sampling, simple random sampling, stratified sampling, cluster sampling, convenience sampling). Specify the locations of sample participants whenever clustered sampling was applied.	3
	6c	Provide information on sample size, along with details of sample size calculation.	3
	6d	Describe how representative the sample is of the study population (or target population if possible), particularly for population-based surveys.	NA
Survey administration	7a	Provide information on modes of questionnaire administration, including the type and number of contacts, the location where the survey was conducted (e.g., outpatient room or by use of online tools, such as SurveyMonkey).	3
	7b	Provide information of survey's time frame, such as periods of recruitment, exposure, and follow-up days.	2-3

	7c	Provide information on the entry process:<br>–>For non-web-based surveys, provide approaches to minimize human error in data entry.<br>–>For web-based surveys, provide approaches to prevent "multiple participation" of participants.	3
Study preparation	8	Describe any preparation process before conducting the survey (e.g., interviewers' training process, advertising the survey).	NA
Ethical considerations	9a	Provide information on ethical approval for the survey if obtained, including informed consent, institutional review board [IRB] approval, Helsinki declaration, and good clinical practice [GCP] declaration (as appropriate).	3
	9b	Provide information about survey anonymity and confidentiality and describe what mechanisms were used to protect unauthorized access.	3
Statistical analysis	10a	Describe statistical methods and analytical approach. Report the statistical software that was used for data analysis.	4
	10b	Report any modification of variables used in the analysis, along with reference (if available).	NA
	10c	Report details about how missing data was handled. Include rate of missing items, missing data mechanism (i.e., missing completely at random [MCAR], missing at random [MAR] or missing not at random [MNAR]) and methods used to deal with missing data (e.g., multiple imputation).	NA
	10d	State how non-response error was addressed.	NA
	10e	For longitudinal surveys, state how loss to follow-up was addressed.	NA
	10f	Indicate whether any methods such as weighting of items or propensity scores have been used to adjust for non-representativeness of the sample.	NA
	10g	Describe any sensitivity analysis conducted.	NA
**Results**			
Respondent characteristics	11a	Report numbers of individuals at each stage of the study. Consider using a flow diagram, if possible.	5
	11b	Provide reasons for non-participation at each stage, if possible.	5
	11c	Report response rate, present the definition of response rate or the formula used to calculate response rate.	5
	11d	Provide information to define how unique visitors are determined. Report number of unique visitors along with relevant proportions (e.g., view proportion, participation proportion, completion proportion).	NA
Descriptive results	12	Provide characteristics of study participants, as well as information on potential confounders and assessed outcomes.	5-7
Main findings	13a	Give unadjusted estimates and, if applicable, confounder-adjusted estimates along with 95% confidence intervals and p-values.	8-13

	13b	For multivariable analysis, provide information on the model building process, model fit statistics, and model assumptions (as appropriate).	8-13
	13c	Provide details about any sensitivity analysis performed. If there are considerable amount of missing data, report sensitivity analyses comparing the results of complete cases with that of the imputed dataset (if possible).	NA
**Discussion**			
Limitations	14	Discuss the limitations of the study, considering sources of potential biases and imprecisions, such as non-representativeness of sample, study design, important uncontrolled confounders.	16
Interpretations	15	Give a cautious overall interpretation of results, based on potential biases and imprecisions and suggest areas for future research.	13-16
Generalizability	16	Discuss the external validity of the results.	13-16
**Other sections**			
Role of funding source	17	State whether any funding organization has had any roles in the survey's design, implementation, and analysis.	16
Conflict of interest	18	Declare any potential conflict of interest.	16
Acknowledgements	19	Provide names of organizations/persons that are acknowledged along with their contribution to the research.	16

S2. Questionnaire on knowledges and practices concerning antibiotics, medication and vaccination

PATIENT CHARACTERISTICS

1. Are you? ☐ A man ☐ A woman
2. What is your age range?
   ☐ 18-29 years
   ☐ 30-39 years
   ☐ 40-49 years
   ☐ 50-59 years
   ☐ 60-69 years
   ☐ 70-79 years
   ☐ 80 years or more
3. What is your marital status?
   ☐ Single
   ☐ Married
   ☐ Common-law
   ☐ Divorced
   ☐ Widowed
4. How many children between 0 and 18 years old live in your household?
   ☐ 0 ☐ 1 ☐ 2 ☐ 3 ☐ 4 ☐ 5
   ☐ 6 ☐ 7 ☐ 8 ☐ 9 ☐ 10 ☐ >10
5. Please specify the age of each of your children:
   1st child: ________ years
   2nd child: ________ years
   3rd child: ________ years
   4th child: ________ years
6. What is your current work situation?
   ☐ On active duty
   ☐ Parental leave
   ☐ In school on training
   ☐ Retired
   ☐ On disability
   ☐ On sick leave of more than 3 months
7. What is the highest degree you have obtained?
   ☐ No diploma
   ☐ College certificate
   ☐ Certificate or vocational studies certificate
   ☐ General, technological or professional bachelor
   ☐ Diploma of bachelor level + 2 years (DUT, BTS, DEUG, School of health or social training...)
   ☐ Second degree diploma (licence, master, master1...)
   ☐ 3rd degree diploma (Master 2, DEA, DESS, Doctorate or graduate school diploma...)
8. What is/was your last statutory category?
   ☐ Category A+
   ☐ Category A
   ☐ Category B
   ☐ Category C
   ☐ Contractual
9. How many inhabitants are there in the commune where you live?
   ☐ Rural municipality
   ☐ 2,000 to 20,000 inhabitants
   ☐ 20,000 to 100,000 inhabitants

☐ 100,000 to 200,000 inhabitants
☐ 200,000 inhabitants and more
☐ Parisian agglomeration

10. In which region do you live?
    ☐ Auvergne-Rhône-Alpes
    ☐ Bourgogne-Franche-Comté
    ☐ Bretagne
    ☐ Centre-Val-de-Loire, Corse
    ☐ Grand Est
    ☐ Hauts-de-France
    ☐ Île-de-France, Normandie
    ☐ Nouvelle Aquitaine
    ☐ Occitanie
    ☐ Pays de la Loire
    ☐ Provence-Alpes-Côte-d'Azur
    ☐ Outremer
11. Do you have a chronic illness (diabetes, asthma...), a disability or a health problem that has been bothering you for at least 6 months and that requires regular care or treatment?
    ☐ Yes ☐ No ☐ Don't know
12. If yes, what type of disease is it?
    a. Respiratory problems (asthma, chronic bronchitis...)
       ☐ Yes ☐ No ☐ Don't know
    b. Disease of the heart, arteries, veins or stroke
       ☐ Yes ☐ No ☐ Don't know
    c. Metabolic disease (diabetes, thyroid, hormones)
       ☐ Yes ☐ No ☐ Don't know
    d. Tumors (cancers, malignant tumors, benign tumors...)
       ☐ Yes ☐ No ☐ Don't know
    e. Mental illness (psychiatric,...)
       ☐ Yes ☐ No ☐ Don't know
    f. Locomotive problems (paraplegia, rheumatism...)
       ☐ Yes ☐ No ☐ Don't know
    g. Other, please specify:________________________
13. Are you currently covered at 100% for a long-term illness by the Health Insurance?
    ☐ Yes ☐ No ☐ Don't know

ANTIBIOTICS

14. Please find below a series of statements about antiobiotics. For each statement, please indicate whether you think it is true or false.
    a. antibiotics are effective in treating viruses
       ☐ Yes ☐ No ☐ Don't know
    b. antibiotics are effective in treating bacteria
       ☐ Yes ☐ No ☐ Don't know
    c. taking antibiotics often can make them less effective
       ☐ Yes ☐ No ☐ Don't know
    d. antibiotics, in general, make it possible to heal more quickly
       ☐ Yes ☐ No ☐ Don't know
15. Do you ever ask your physician for antibiotics for yourself or your family?
    ☐ Yes ☐ No
16. Do you ever stop your antibiotic treatment as soon as you feel better?

☐ Yes ☐ No

17. Do you ever reuse antibiotics left in your medicine cabinet?
    a. For you
       ☐ Yes ☐ No
    b. For your child(ren)
       ☐ Yes ☐ No
    c. For your entourage
       ☐ Yes ☐ No
18. Who do you trust first to give you information on the proper use of antibiotics? (Ranking from 1 to 7 in order of confidence)
    ___ general practitioner/paediatrician
    ___ prevention doctor (occupational physician)
    ___ nurse
    ___ pharmacist
    ___ family
    ___ traditional media (TV, radio, press...)
    ___ public authority website (French Health Insurance, French Public Health...)

MEDICATION

19. How many different medications do you currently take per day? We are talking about the number of different products and not the number of doses (tablets, capsules, sachets...)
    ☐ 0 ☐ 1 ☐ 2 ☐ 3 ☐ 4 ☐ > 5
20. Do you feel you are taking too much medication?
    ☐ Yes ☐ No
21. Have you ever discussed with your doctor how many medications you are taking?
    ☐ Yes, at your initiative
    ☐ Yes, at the initiative of your doctor
    ☐ No
22. Have you ever discussed with your pharmacist the number of medications you are taking?
    ☐ Yes, at your initiative
    ☐ Yes, at the initiative of your pharmacist
    ☐ No
23. Are you usually helped to take your medication by?
    a. a nurse ☐ Yes ☐ No
    b. a relative (family, friend, neighbor...) ☐ Yes ☐ No
    c. a home help (life auxiliary, housekeeper...) ☐ Yes ☐ No

24. In the past month, have you taken any medications to help you sleep?
    ☐ Yes, every day or almost every day
    ☐ Yes, several times a week
    ☐ Yes, once a week
    ☐ Yes, less often
    ☐ No
25. In the past month, have you taken any medication to help manage your anxiety and/or stress?
    ☐ Yes, every day or almost every day
    ☐ Yes, several times a week
    ☐ Yes, once a week

☐ Yes, less often
☐ No

26. If yes to question 24 or 25, has your doctor or pharmacist warned you about the risks of dependence linked to the duration of consumption of this type of medication?
    ☐ Yes, my doctor
    ☐ Yes, my pharmacist
    ☐ No
27. If yes to question 24 or 25, have you tried non-medicinal methods to help you sleep better or manage your anxiety better (psychotherapy, improved lifestyle, sophrology, sports, meditation, etc.)?
    ☐ Yes, I did
    ☐ Yes, I wanted to, but haven't done it yet
    ☐ Yes, I want to but I can't do it
    ☐ No

VACCINATION

28. Do you think you are up to date with your vaccinations?
    ☐ Yes ☐ No ☐ Don't know
29. Please read the following statements about vaccination. For each statement, please indicate whether you think it is true or false.
    a. it is useless to be vaccinated against a disease that has long since disappeared in our country
       ☐ Yes ☐ No ☐ Don't know
    b. it is better to develop one's own immune defenses by having the disease than by being vaccinated
       ☐ Yes ☐ No ☐ Don't know
    c. vaccines are effective and useful
       ☐ Yes ☐ No ☐ Don't know
    d. vaccines cause serious side effects
       ☐ Yes ☐ No ☐ Don't know

30. Would you be interested in receiving more information about?
    a. the vaccination calendar (mandatory and recommended vaccines)
       ☐ Yes ☐ No
    b. the composition of the vaccines
       ☐ Yes ☐ No
    c. the current state of scientific knowledge about vaccines
       ☐ Yes ☐ No
    d. Recommended vaccines for foreign travel
       ☐ Yes ☐ No
31. Who do you trust most to give you information about vaccination? (Ranking from 1 to 7 in order of confidence)
    ___ general practitioner/paediatrician
    ___ prevention doctor (occupational physician)
    ___ nurse
    ___ pharmacist
    ___ family
    ___ traditional media (TV, radio, press...)
    ___ public authority website (French Health Insurance, French Public Health...)

32. Do you think you would get vaccinated more easily if...?

a. it was possible to be vaccinated at the workplace

☐ Yes ☐ No

b. pharmacists had the possibility to do it in their dispensary

☐ Yes ☐ No

c. the cost to you was less

☐ Yes ☐ No

d. the vaccines were free

☐ Yes ☐ No

e. the vaccines were mandatory

☐ Yes ☐ No

f. you receive complete and personalized information on vaccines

☐ Yes ☐ No

g. you receive information about epidemics and viruses

☐ Yes ☐ No

h. you receive information about your vaccination history (vaccination booklet)

☐ Yes ☐ No

S3. Multiple Correspondence Analysis (MCA) results

- **Variables**

Table S1. Variables considered in the MCA

	Frequency (n)	Proportion of respondents (%)
Gender:		
- Women	12785	58.9
- Men	8938	41.1
Age in 10-year steps:		
- 18-29 years old	240	1.1
- 30-39 years old	1483	6.8
- 40-49 years old	3475	16.0
- 50-59 years old	5008	23.1
- 60-69 years old	7309	33.6
- 70-79 years old	3681	17.0
- 80 years old and older	527	2.4
Marital status:		
- Single	2592	12.0
- Divorced	2457	11.3
- Married	12794	58.9
- Civil partnership	1240	5.7
- Common-law	1524	7.0
- Widowed	1116	5.1
Level of education:		
- No diploma	587	23.9
- Baccalaureate	5198	10.5
- Brevet of colleges	2274	19.6
- Certificate of professional competence or Certificate of professional studies	4253	16.4
- Diploma of Bac + 2 level	3565	16.4
- 2nd degree diploma	3562	10.5
- 3rd degree diploma	2284	2.7
Last professional category:		
- A+	2600	12.0
- A	4236	19.5
- B	8705	40.1
- C	5802	26.7
- Contractual	380	1.7

Region of residence:		
- Auvergne Rhône-Alpes	2307	10.6
- Bourgogne Franche-Comté	1010	4.6
- Bretagne	941	4.3
- Centre Val-de-Loire	1237	5.7
- Corse	186	0.8
- DOM	1129	5.2
- Grand-Est	1867	8.6
- Hauts-de-France	1433	6.6
- Ile-de-France	3318	15.3
- Normandie	884	4.1
- Nouvelle Aquitaine	2599	12.0
- Occitanie	2162	10.0
- Pays-de-la-Loire	1228	5.7
- Provence-Alpes-Côte-d'Azur	1422	6.5
Chronic disease:		
- At least one chronic disease	6734	31.0
- No chronic disease	14989	69.0
Professional status:		
- At school, training, internship	50	0.2
- Retired	10709	49.3
- On parental leave	57	0.3
- Active working	10494	48.3
- On sick leave for over 3 months	321	1.5
- On disability	92	0.4

- **Inertia**

The total inertia is about 5.125.

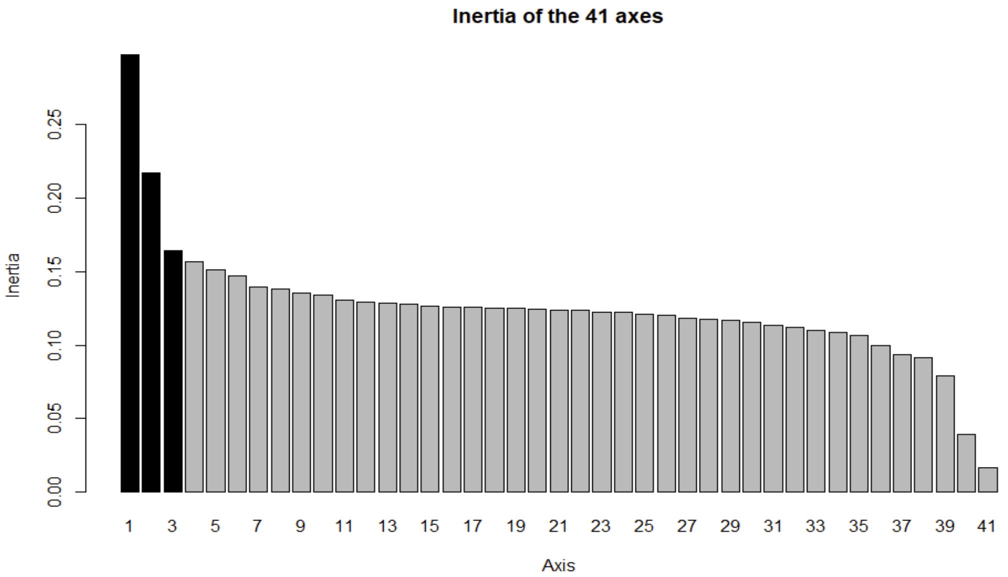

Figure S1. Inertia of the 41 axes

- **Percentage of explained variances of the 10 first axes**

The first axis explained about 5.8%, and the two first axis explained about 10.0% of the observed variability of the sample.

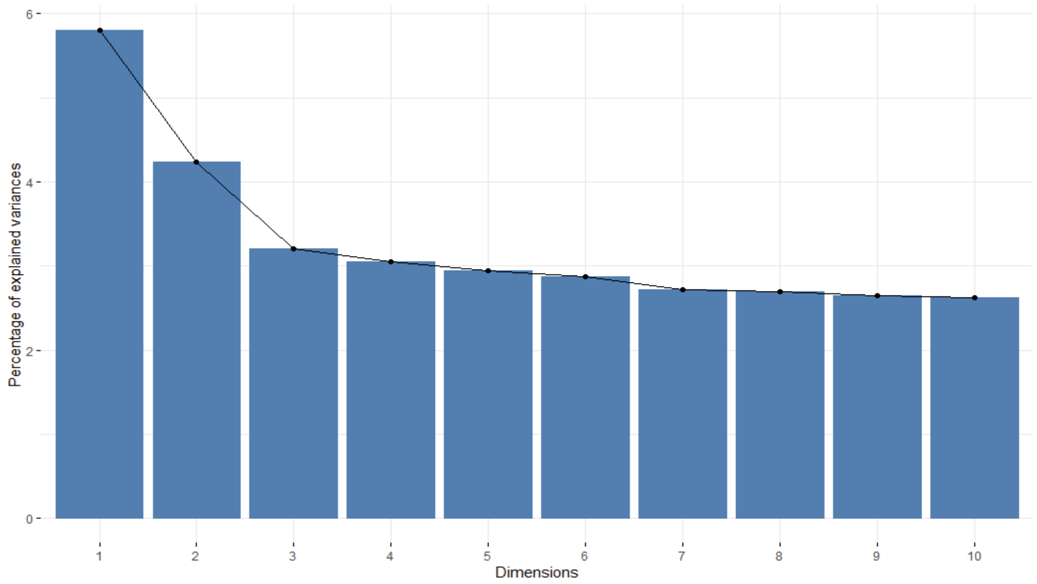

Figure S2. Percentage of explained variances of the 10 first axes

- **Correlation circle of the variables**

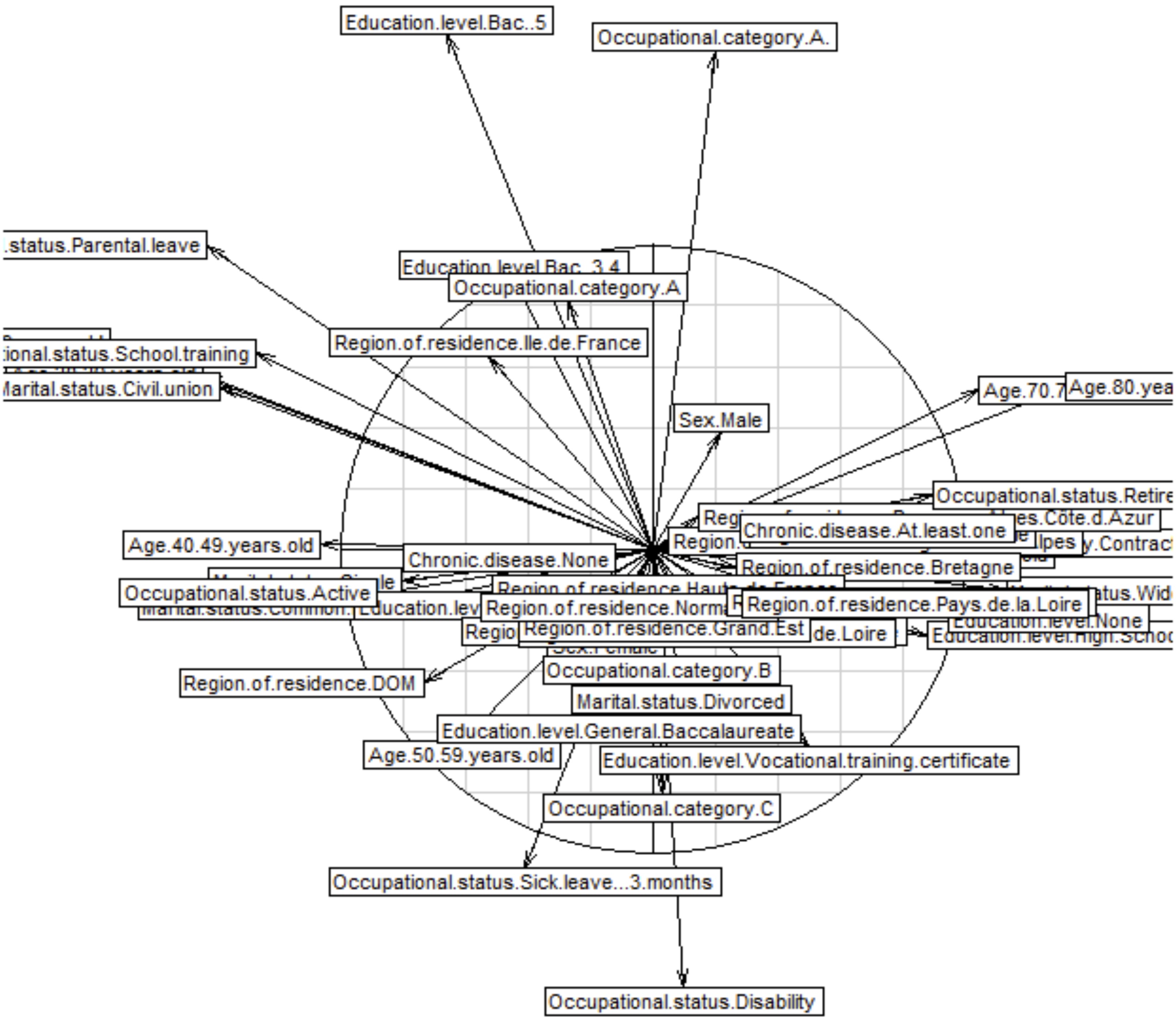

Figure S3. Correlation circle of variables

- **Repartition of the responses according to the first axis**

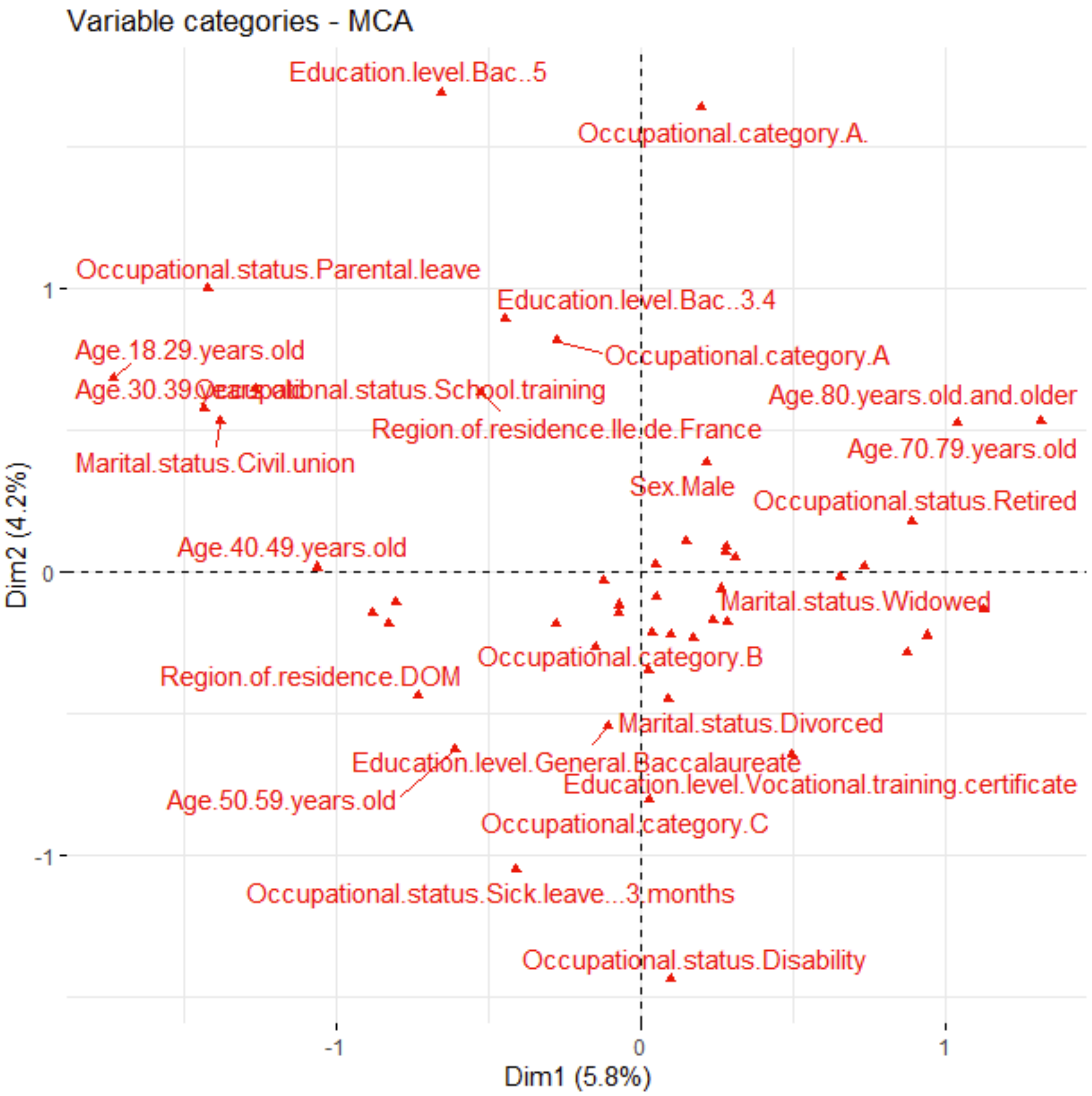

Figure S4. Repartition of the responses according to the first axis

- **MCA based on socio-demographic variables**

The MCA based on socio-demographic variables consisted of 41 factorial axes for a total inertia about 5.125. The two first axis explained only 10.0% of the observed variability of the sample. The low proportion of explained variability is due to inter-correlations between the socio-demographic variables. However, the two first factorial axis revealed a two-fold structure.

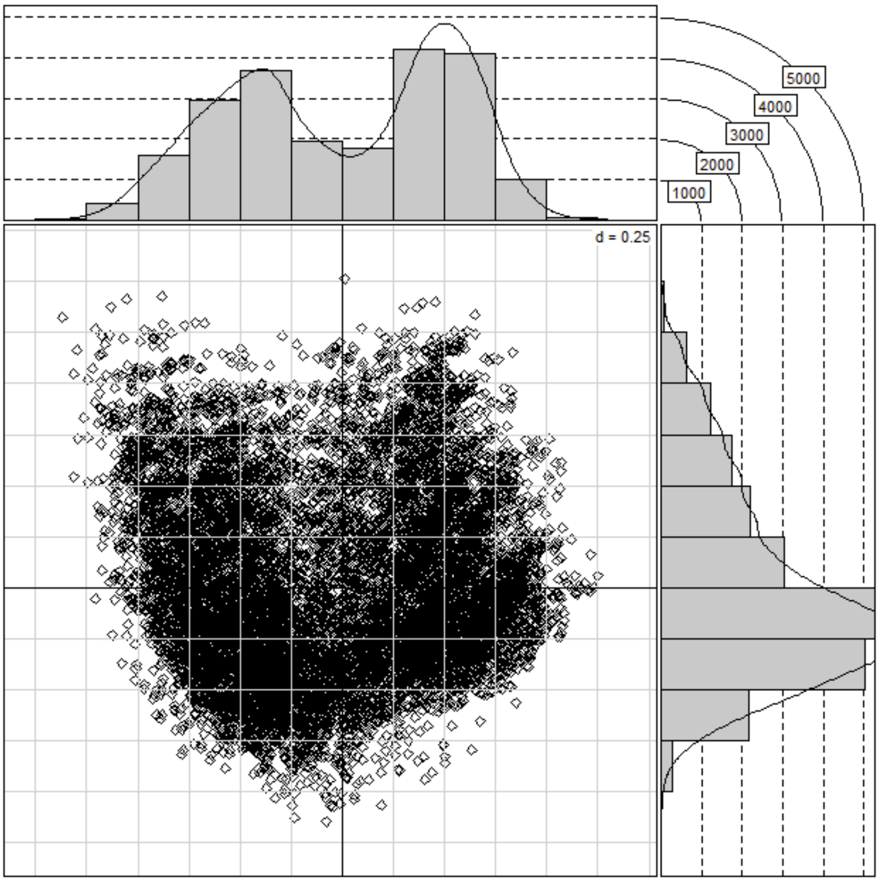

Figure S5. Projection of the 21 723 included respondents on the two first factorial axis and display of their density by steps of 1000 respondents on x-axis on the upper side, and y-axis on the right side.

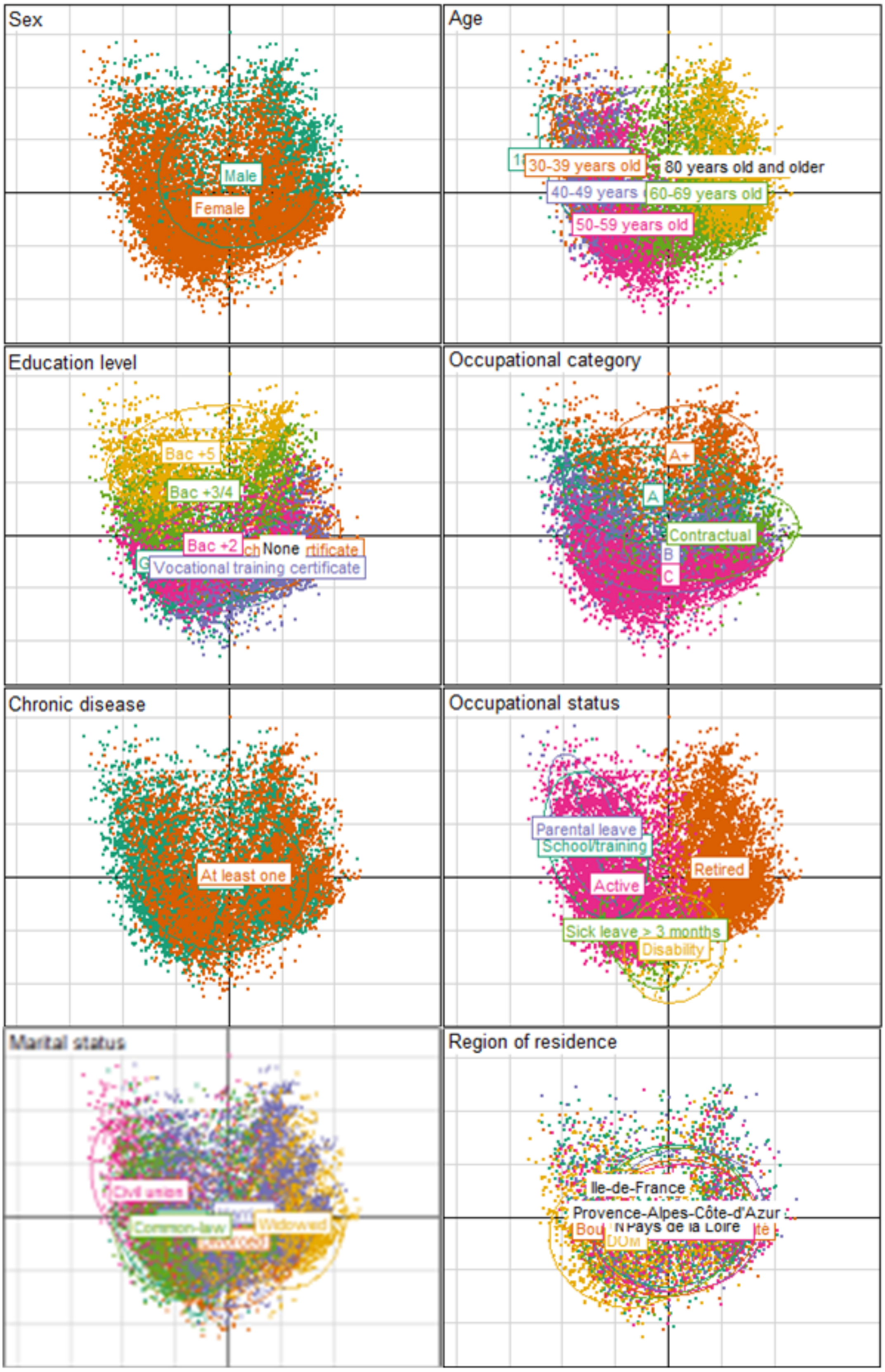

Figure S6. Projection of the distribution of the modalities of each socio-demographic variable on the included respondents' positions on the two first factorial axis.

Among the least overlapping groups of modalities, age and occupational status were the most explanatory variables of the MCA structure. While the y-axis was structured depending of the occupational category, the age range was the most explanatory variable of the respondents repartition on the x-axis. The respondents under 60 years old were on the left while the over 60 years old are on the right. The most marked dichotomy was observed for the occupational status with the workers and the retired respectively on the left and on the right. Workers group corresponds to active persons, parental leave, school training, stick leave > 3 months whereas retired group corresponds to persons retired from the public service whatever their age and cause. These two groups based on the occupational status were considered as the most dichotomous of the sample respondents. The results of this analysis did not identify other socio-demographic variables that were sufficiently contributive to manage the statistical analysis of the survey questions to another specific subgroup analysis.

S4. Repartition of the respondents according to the age and territorial repartition

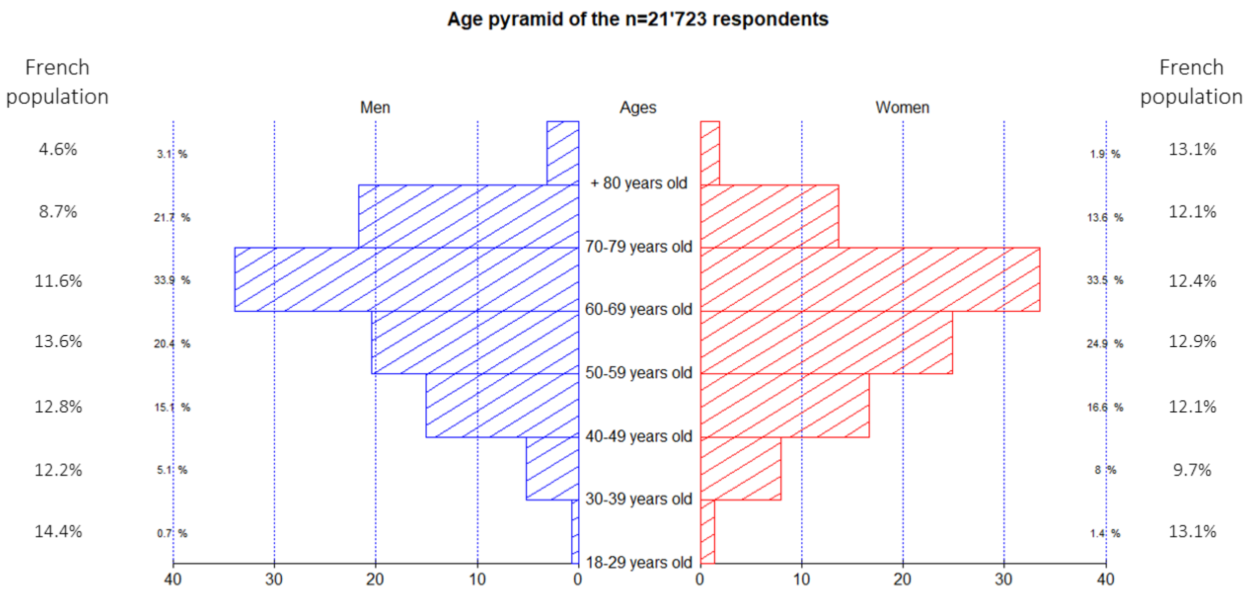

Figure S7. Repartition of the respondents and the french population according to age

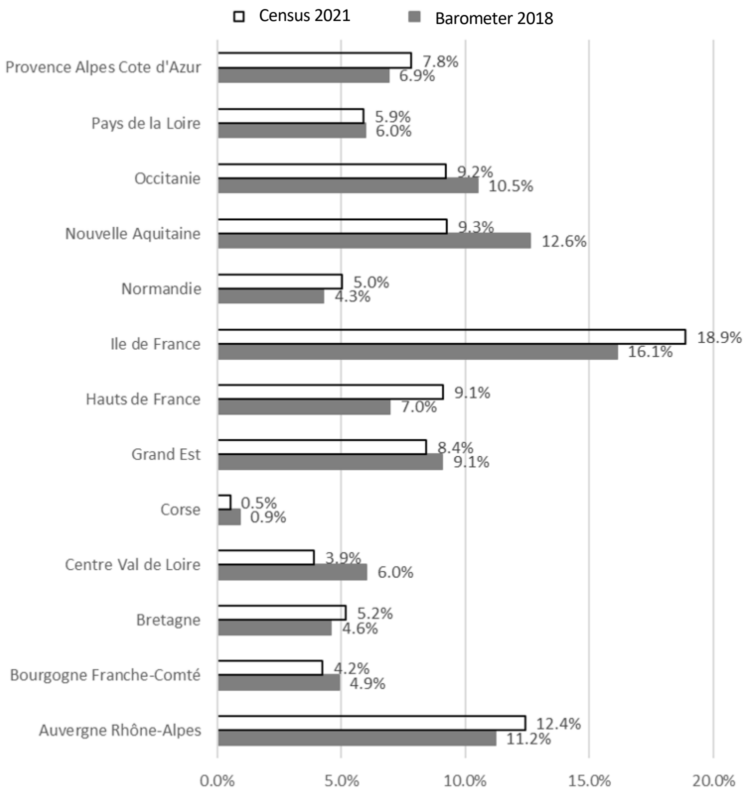

Figure S8. Repartition of the respondents and the french population according to territorial repartition

S5. Knowledges, attitudes and practices related to polymedication, antibiotics, and vaccination among public service workers and retirees

Table S2. Knowledges, attitudes and practices related to antibiotics among public service workers and retirees. The results are expressed as n (%). n: number of respondents, %: percentage.

Question	All (N=21723)	Active (N=11014)	Retired (N=10709)	p-value
**Antibiotics are effective in treating viruses**				< 0.0001
Yes	3923 (18.06)	1874 (17.00)	2049 (19.10)	
No	15521 (71.45)	8143 (73.90)	7378 (68.90)	
Don't know	2279 (10.49)	997 (9.10)	1282 (12.00°	
**Antibiotics are effective in treating bacteria**				< 0.0001
Yes	16116 (74.19)	8531 (77.50)	7585 (70.80)	
No	2693 (12.4)	1198 (10.90)	1495 (14.00)	
Don't know	2914 (13.41)	1285 (11.70)	1629 (15.20)	
**Taking antibiotics often can make them less effective**	19849 (91.37)	10231 (92.90)	9618 (89.80)	< 0.0001
Yes	762 (3.51)	323 (2.90)	439 (4.10)	
No	1112 (5.12)	460 (4.20)	652 (6.10)	
Don't know	19849 (91.37)	10231 (92.90)	9618 (89.80)	
**Antibiotics, in general, make it possible to heal more quickly**				< 0.0001
Yes	10771 (49.58)	4824 (43.80)	5947 (55.50)	
No	7842 (36.10)	4766 (36.10)	3076 (28.70)	
Don't know	3110 (14.32)	1424 (12.90)	1686 (15.70)	
**Do you ever ask your physician for antibiotics for yourself or your family?**				< 0.0001
Yes	2473	1371 (12.40)	1102 (10.30)	
No	19250	9643 (87.60)	9607 (89.70)	
**Do you ever stop your antibiotic treatment as soon as you feel better?**				< 0.0001
Yes	2628	1601 (14.50)	1027 (9.60)	

No	19095	9413 (85.50)	9682 (90.40)	
**Do you ever use antibiotics left in your medicine cabinet?**				< 0.0001
Yes	4555	2809 (25.50)	1746 (16.30)	
No	17168	8205 (74.50)	8963 (83.70)	
**Do you ever use antibiotics left in your medicine cabinet for you?**				< 0.0001
Yes	4463	2748 (25.00)	1715 (16.00)	
No	17260	8266 (75.00)	8994 (84.00)	
**Do you ever use antibiotics left in your medicine cabinet for your child(ren)?**				< 0.0001
Yes	701	585 (5.30)	116 (1.10)	
No	21022	10429 (94.70)	10593 (98.90)	
**Do you ever use antibiotics left in your medicine cabinet for your entourage?**				< 0.0001
Yes	973	698 (6.30)	275 (2.60)	
No	20750	10316 (93.70)	10434 (97.40)	
**Who do you trust first to give you information on the proper use of antibiotics?**				
General practitioner/paediatrician	20223	10211 (92.70)	10012 (93.50)	0.0102
Prevention doctor (occupational physician)	168	74 (0.70)	94 (0.90)	< 0.0001
Nurse	76	35 (0.30)	41 (0.40)	0.4718
Pharmacist	771	429 (3.90)	342 (3.20)	0.0001
Family	76	53 (0.50)	23 (0.20)	< 0.0001
Traditional media (TV, radio, press...)	73	38 (0.30)	35 (0.30)	< 0.0001
Public authority website (French Health Insurance, French Public Health...)	336	174 (1.60)	162 (1.50)	< 0.0001

Table S3. Knowledges, attitudes and practices related to polymedication among public service workers and retirees. The results are expressed as n (%). n: number of respondents, %: percentage.

Question	All (N=21723)	Active (N=11014)	Retired (N=10709)	p-value
**How many different medications do you currently take per day?**				< 0.0001
0	8151	5614 (51.00)	2537 (23.70)	
1 to 3	9998	4444 (40.30)	5554 (51.90)	
4 to 5	2318	623 (5.70)	1695 (15.80)	
5+	1256	333 (3.00)	923 (8.60)	
**Do you feel you are taking too much medication?**				< 0.0001
Yes	3521	1465 (13.30)	2056 (19.20)	
No	10051	3935 (35.70)	6116 (57.10)	
Don't know	8151	5614 (51.00)	2537 (23.70)	
**Have you ever discussed with your doctor how many medications you are taking?**				< 0.0001
Yes, at the initiative of your doctor	2086	601 (5.50)	1485 (13.90)	
Yes, at your initiative	4606	1593 (14.50)	3013 (28.10)	
No	6880	3206 (29.10)	3674 (34.30)	
**Have you ever discussed with your pharmacist the number of medications you are taking?**				< 0.0001
Yes, at the initiative of your pharmacist	423	149 (1.40)	274 (2.60)	
Yes, at your initiative	1710	578 (5.20)	1132 (10.60)	
No	11439	4673 (42.40)	6766 (63.20)	
**Are you usually helped to take your medication by a nurse?**				< 0.0001
Yes	135	50 (0.50)	85 (0.80)	
No	13437	5350 (48.60)	8087 (75.50)	

**Are you usually helped to take your medication by a relative (family, friend, neighbor...)?**				< 0.0001
Yes	301	96 (0.90)	205 (1.90)	
No	13271	5304 (48.20)	7967 (74.40)	
**Are you usually helped to take your medication by a home help (life auxiliary, housekeeper...)?**				< 0.0001
Yes	32	12 (0.10)	20 (0.20)	
No	13540	5388 (48.90)	8152 (76.10)	
**In the past month, have you taken any medications to help you sleep?**				< 0.0001
Yes, every day or almost every day	1383	620 (5.60)	763 (7.10)	
Yes, several times a week	820	471 (4.30)	349 (3.30)	
Yes, once a week	451	222 (2.00)	229 (2.10)	
Yes, less often	1775	928 (8.40)	847 (7.90)	
No	17294	8773 (79.70)	8521 (79.60)	
**In the past month, have you taken any medication to help manage your anxiety and/or stress?**				< 0.0001
Yes, every day or almost every day	1823	1029 (9.30)	794 (7.40)	
Yes, several times a week	721	459 (4.20)	262 (2.40)	
Yes, once a week	382	214 (1.90)	168 (1.60)	
Yes, less often	1719	969 (8.80)	750 (7.00)	
No	17078	8343 (75.70)	8735 (81.60)	
**Has your doctor warned you about the risks of dependence linked to the duration of consumption of this type of medication?**				< 0.0001
Yes	3839	2001 (18.20)	1838 (17.20)	
No	2328	1286 (11.70)	1042 (70.00)	
**Has your pharmacist warned you about the risks of dependence linked to the duration of consumption of this type of medication?**				< 0.0001

Yes	1176	678 (6.20)	498 (4.70)	
No	4991	2609 (23.70)	2382 (22.20)	
**Have you tried non-medicinal methods to help you sleep better or manage your anxiety better (psychotherapy, improved lifestyle, sophrology, sports, meditation, etc.)?**				< 0.0001
Yes, I did	3933	2235 (20.30)	1698 (15.90)	
Yes, I wanted to, but haven't done it yet	426	248 (2.30)	178 (1.70)	
Yes, I want to but I can't do it	396	235 (2.10)	161 (1.50)	
No	8858	569 (5.20)	843 (7.90)	

Table S4. Knowledges, attitudes and practices related to vaccination among public service workers and retirees. The results are expressed as n (%). n: number of respondents, %: percentage.

$	All (N=21723)	Active (N=11014)	Retired (N=10709)	p-value
**Do you think you are up to date with your vaccinations?**				< 0.0001
Yes	16242	8703 (79.00)	7539 (70.40)	
No	2798	1155 (10.50)	1643 (15.30)	
No answer	2683	1156 (10.50)	1527 (14.30)	
**It is useless to be vaccinated against a disease that has long since disappeared in our country**				< 0.0001
Yes	2936	1187 (10.80)	1749 (16.30)	
No	15976	8696 (79.00)	7280 (68.00)	
No answer	2811	1131 (10.30)	1680 (15.70)	
**It is better to develop one's own immune defenses by having the disease than by being vaccinated**				0.0001
Yes	2544	1305 (11.80)	1239 (0)	
No	15342	7883 (71.60)	7459 (69.70)	
No answer	3837	1826 (16.60)	2011 (18.80)	
**Vaccines are effective and useful**				0.1041
Yes	19175	9680 (87.90)	9495 (88.70)	
No	852	460 (4.20)	392 (3.70)	
No answer	1696	874 (7.90)	822 (7.70)	
**Vaccines cause serious side effects**				< 0.0001
Yes	4941	2931 (26.60)	2010 (18.80)	
No	9770	4872 (44.20)	4898 (45.70)	
No answer	7012	3211 (29.20)	3801 (35.50)	

**Would you be interested in receiving more information about the vaccination calendar (mandatory and recommended vaccines)?**				< 0.0001
Yes	14055	7422 (67.40)	6633 (61.90)	
No	7668	3592 (32.60)	4076 (38.10)	
**Would you be interested in receiving more information about the composition of the vaccines?**				< 0.0001
Yes	10278	5562 (50.50)	4716 (44.00)	
No	11445	5452 (49.50)	5993 (56.00)	
**Would you be interested in receiving more information about the current state of scientific knowledge about vaccines?**				< 0.0001
Yes	11050	5753 (52.20)	5297 (49.50)	
No	10673	5261 (47.80)	5412 (50.50)	
**Would you be interested in receiving more information about recommended vaccines for foreign travel?**				< 0.0001
Yes	11308	6009 (54.60)	5299 (49.50)	
No	10415	5005 (45.40)	5410 (50.50)	
**Who do you trust most to give you information about vaccination?**				
General practitioner/paediatrician	20150	10163 (92.30)	9987 (93.30)	0.0011
Prevention doctor (occupational physician)	331	170 (1.50)	161 (1.50)	0.0001
Nurse	91	53 (0.50)	38 (0.40)	0.0006
Pharmacist	236	122 (1.10)	114 (1.10)	< 0.0001
Family	174	119 (1.10)	55 (0.50)	< 0.0001
Traditional media (TV, radio, press...)	203	120 (1.10)	83 (0.80)	< 0.0001
Public authority website (French Health Insurance, French Public Health...)	538	267 (2.40)	271 (2.50)	< 0.0001
**Do you think you would get vaccinated more easily if it was possible to be vaccinated at the workplace?**				< 0.0001
Yes	11362	6684 (60.70)	4678 (43.70)	

No	10361	4330 (39.30)	6031 (56.30)	
**Do you think you would get vaccinated more easily if pharmacists had the possibility to do it in their dispensary?**				0.2591
Yes	11392	5818 (52.80)	5574 (52.00)	
No	10331	5196 (47.20)	5135 (48.00)	
**Do you think you would get vaccinated more easily if the cost to you was less?**				< 0.0001
Yes	10007	5386 (48.90)	4621 (43.20)	
No	11716	5628 (51.10)	6088 (56.80)	
**Do you think you would get vaccinated more easily if the vaccines were free?**				< 0.0001
Yes	11528	6013 (54.60)	5515 (51.50)	
No	10195	5001 (45.40)	5194 (48.50)	
**Do you think you would get vaccinated more easily if the vaccines were mandatory?**				< 0.0001
Yes	9718	4670 (42.40)	5048 (47.10)	
No	12005	6344 (57.60)	5661 (52.90)	
**Do you think you would get vaccinated more easily if you receive complete and personalized information on vaccines?**				< 0.0001
Yes	12821	6770 (61.50)	6051 (56.50)	
No	8902	4244 (38.50)	4658 (43.50)	
**Do you think you would get vaccinated more easily if you receive information about epidemics and viruses?**				0.0011
Yes	12722	6332 (57.50)	6390 (59.70)	
No	9001	4682 (42.50)	4319 (40.30)	
**Do you think you would get vaccinated more easily if you receive information about your vaccination history (vaccination booklet)?**				< 0.0001
Yes	14933	7850 (71.30)	7083 (66.10)	
No	6790	3164 (28.70)	3626 (33.90)	
